# Genome-Wide Analysis of Targets for Post-Transcriptional Regulation by Rsm Proteins in *Pseudomonas putida*

**DOI:** 10.3389/fmolb.2021.624061

**Published:** 2021-02-22

**Authors:** Óscar Huertas-Rosales, Manuel Romero, Kok-Gan Chan, Kar-Wai Hong, Miguel Cámara, Stephan Heeb, Laura Barrientos-Moreno, María Antonia Molina-Henares, María L. Travieso, María Isabel Ramos-González, Manuel Espinosa-Urgel

**Affiliations:** ^1^ Department of Environmental Protection, Estación Experimental del Zaidín, CSIC, Granada, Spain; ^2^ National Biofilms Innovation Centre, Biodiscovery Institute and School of Life Sciences, University of Nottingham, Nottingham, United Kingdom; ^3^ Division of Genetics and Molecular Biology, Institute of Biological Sciences, Faculty of Science, University of Malaya, Kuala Lumpur, Malaysia; ^4^ International Genome Centre, Jiangsu University, Zhenjiang, China

**Keywords:** RNA-binding proteins, global regulation, biofilm, rhizosphere, amino acid metabolism, c-di-GMP signaling

## Abstract

Post-transcriptional regulation is an important step in the control of bacterial gene expression in response to environmental and cellular signals. *Pseudomonas putida* KT2440 harbors three known members of the CsrA/RsmA family of post-transcriptional regulators: RsmA, RsmE and RsmI. We have carried out a global analysis to identify RNA sequences bound *in vivo* by each of these proteins. Affinity purification and sequencing of RNA molecules associated with Rsm proteins were used to discover direct binding targets, corresponding to 437 unique RNA molecules, 75 of them being common to the three proteins. Relevant targets include genes encoding proteins involved in signal transduction and regulation, metabolism, transport and secretion, stress responses, and the turnover of the intracellular second messenger c-di-GMP. To our knowledge, this is the first combined global analysis in a bacterium harboring three Rsm homologs. It offers a broad overview of the network of processes subjected to this type of regulation and opens the way to define what are the sequence and structure determinants that define common or differential recognition of specific RNA molecules by these proteins.

## Introduction

By coordinating the expression of a large number of genes, global regulatory networks allow bacteria to adjust their physiology according to environmental stimuli, changes in their lifestyle, or in nutritional status (Ishihama, 2010; Coggan and Wolfgang, 2012; Shis et al., 2018). Transcriptional regulators and sigma factors influencing the expression of different sets of bacterial genes have been widely studied for decades, starting shortly after the postulates of Jacob and Monod on operons, operators and messenger RNA were put forward (Jacob and Monod, 1961). A second instance of protein expression regulation, namely post-transcriptional modulation affecting mRNA stability, structure or translation, mediated by proteins or small non-coding RNAs, has gained increasing attention in the past 2 decades, but is still less well studied (Romeo et al., 2013; Van Assche et al., 2015; Hör et al., 2018). Among the post-transcriptional regulators identified in prokaryotes, the CsrA/RsmA family of proteins seems to be widely conserved in many bacteria and, in some cases, more than one member of this family are present in a single species (Reimmann et al., 2005; Morris et al., 2013; Huertas-Rosales et al., 2016; Ferreiro et al., 2018). These small RNA-binding proteins generally function as negative effectors of translation by binding to the 5′untranslated region of target mRNAs, close to or overlapping with the ribosome binding site (Baker et al., 2007; Yakhnin et al., 2007), or by causing premature transcription termination through alterations of the RNA structure that lead to the exposure of a Rho binding site (Figueroa-Bossi et al., 2014). However, they can also influence mRNA stability in a positive way, for example, by masking RNase E cleavage sites (Yakhnin et al., 2013). CsrA/RsmA proteins can interact with two RNA motifs, with a strong preference for 5′-RUACARGGAUGU-3′ consensus sequences located in the loops of short RNA hairpins (Dubey et al., 2005; Duss et al., 2014). Small non-coding RNAs (sRNA) containing multiple binding motifs play an opposing regulatory role by sequestering the CsrA/RsmA proteins, thus limiting their access to target mRNAs (Romeo, 1998; Kay et al., 2005; Sonnleitner and Haas, 2011; Janssen et al., 2018). In *Pseudomonas*, expression of these regulatory RNAs is controlled by the two-component system GacS/GacA (Brencic et al., 2009), in response to as yet not well-defined signal(s).

CsrA/RsmA proteins play a global role in modulating gene expression (Romeo, 1998; Lawhon et al., 2003; Brencic and Lory, 2009; Romeo et al., 2013; Romero et al., 2018). The functions identified as being under this type of regulation in different bacteria include carbohydrate metabolism and storage (Sabnis et al., 1995; Yang et al., 1996; Pannuri et al., 2016), synthesis of flagellar components (Yakhnin et al., 2007, Yakhnin et al., 2013), the production of secondary metabolites (Sonnleitner and Haas, 2011; Morris et al., 2013), quorum sensing signaling (Lenz et al., 2005), or the expression of virulence factors (Heroven et al., 2012; Sterzenbach et al., 2013; Vakulskas et al., 2015; Ferreiro et al., 2018). Global analyses have been done in bacteria harboring one CsrA/RsmA family protein to identify elements in the signaling and regulatory network associated to them (Lawhon et al., 2003; Sowa et al., 2017) or to othologous elements (Romero et al., 2018).

In the plant-root colonizing, beneficial bacterium *Pseudomonas putida* KT2440, three genes have been identified that encode post-transcriptional regulators belonging to the CsrA/RsmA family. These proteins (RsmA, RsmE and RsmI), have opposing effects on surface motility and biofilm formation; deletion of the three genes abolishes swarming motility and stimulates bacterial attachment and biofilm formation, although the biofilms formed by a triple *rsm* mutant are more labile and easily dispersed than wild type biofilms (Huertas-Rosales et al., 2016). These alterations are associated with changes in the expression of some components of the extracellular matrix of biofilms (Huertas-Rosales et al., 2016) and with increased levels of the intracellular second messenger cyclic diguanylate (c-di-GMP) (Huertas-Rosales et al., 2017). The three proteins were found to bind specific motifs in the leader sequence and translation initiation region of the mRNA of *cfcR* (Huertas-Rosales et al., 2017), which encodes a response regulator with diguanylate cyclase activity (Matilla et al., 2011; Ramos-González et al., 2016). Although the binding affinity was different for each Rsm protein, deletion of any single one of the three genes had no significant influence on expression of *cfcR* (Huertas-Rosales et al., 2017), indicating the existence of some functional redundancy between RsmA, RsmE and RsmI. Based on sequence similarity with related strains, the putative antagonistic sRNAs RsmY and RsmZ could also be identified in KT2440 (Huertas-Rosales et al., 2016). Still, little is known about the binding specificities of these proteins in *P. putida*.

To further understand the importance of these proteins in signal transduction and regulation of global gene expression in *P. putida*, we have used a high-throughput approach to identify RNA sequences bound by Rsm proteins. Our data indicate that a significant number of genes are susceptible of being modulated at the post-transcriptional level by these proteins, and support the existence of a certain degree of functional overlap between the three Rsm homologs. This approach has enabled us to gain new insights into the biological function of these post-transcriptional regulators in *P. putida*, including their role in some metabolic processes and bacterial fitness in the plant root environment.

## Materials and Methods

### Bacterial Strains, Culture Media and Growth Conditions

The bacterial strains, plasmids and oligonucleotides used in this study are listed in Table 1. *Pseudomonas putida* KT2440 is a plasmid-free derivative of *P. putida* mt-2 (Regenhardt et al., 2002). *Pseudomonas putida* strains were grown at 30°C, in rich LB medium (Lennox, 1955), M9 or M8 defined medium (Sambrook and Russell, 2001) supplemented with 1 mM MgSO_4_, 6 mg/L ammonium ferric citrate and trace metals as described previously (Yousef-Coronado et al., 2008). Unless otherwise indicated, glucose (20 mM) or sodium citrate (15 mM) were used as carbon sources. *Escherichia coli* strains were grown at 37°C in LB. When appropriate, antibiotics were added to the medium at the following final concentrations (µg/ml): ampicillin (Ap) 100; kanamycin (Km) 25; streptomycin (Sm) 50 (*E. coli*) or 100 (*P. putida*); (Gm) gentamycin 50; tetracycline (Tc) 10 or 20. Cell growth was followed by measuring optical density at 660 nm (OD_660_).

**TABLE 1:** Bacterial strains, plasmids and oligonucleotides used in this work.

Strains	Genotype/relevant characteristics	References/source
*P. putida*		
KT2440	Wild-type, pWWO-free derivative of *P. putida* mt-2	PRCC^a^
ΔI	Null *rsmI* derivative of KT2440	10
ΔE	Null *rsmE* derivative of KT2440	10
ΔA	Null *rsmA* derivative of KT2440	10
ΔIE	Double null *rsmI, rsmE* derivative of KT2440	10
ΔIA	Double null *rsmI*, *rsmA* derivative of KT2440	10
ΔEA	Double null *rsmE*, *rsmA* derivative of KT2440	10
ΔIEA	Triple null *rsmI*, *rsmE*, *rsmA* derivative of KT2440	10
KT2440-miniTn7-Km	Km^R^, miniTn7Km-tagged derivative of KT2440	This work
KT2440-miniTn7-Sm	Sm^R^, miniTn7Sm-tagged derivative of KT2440	This work
ΔA-miniTn7-Sm	Sm^R^, miniTn7Sm-tagged derivative of Δ*rsmA*	This work
ΔE-miniTn7-Sm	Sm^R^, miniTn7Sm-tagged derivative of Δ*rsmE*	This work
ΔI-miniTn7-Sm	Sm^R^, miniTn7Sm-tagged derivative of Δ*rsmI*	This work
ΔEA-miniTn7-Sm	Sm^R^, miniTn7Sm-tagged derivative of Δ*rsmEA*	This work
ΔIA-miniTn7-Sm	Sm^R^, miniTn7Sm-tagged derivative of Δ*rsmIA*	This work
ΔIE-miniTn7-Sm	Sm^R^, miniTn7Sm-tagged derivative of Δ*rsmIE*	This work
ΔIEA-miniTn7-Sm	Sm^R^, miniTn7Sm-tagged derivative of Δ*rsmIEA*	This work
*E. coli*		
AKN63 (pBK-miniTn7-ΩSm1)	Ap^R^, Sm^R^, strain for delivery of miniTn7Sm	42
Pir1 (pUC18R6KT-mini-Tn7Km)	Ap^R^, Km^R^, strain for delivery of miniTn7Km	Addgene
SM10 *λpir* (pUX-BF13)	Ap^R^, RP4 transfer functions and miniTn7 transposase helper	42
DH5α (pRK600)	Cm^R^, helper for conjugation	PRCC
Plasmids		
pME6032-*rsmA*	Tc^R^, derivative of pME6032 for expression of RsmA-His_6_	10
pME6032-*rsmE*	Tc^R^, derivative of pME6032 for expression of RsmE-His_6_	10
pME6032-*rsmI*	Tc^R^, derivative of pME6032 for expression of RsmI-His_6_	10
Oligonucleotides	Sequence (5´→3′)^b^	Use
PT7*rpoS*Fw	TTT​TCT​GCA​G**TAA​TAC​GAC​TCA​CTA​TAG​G**CTC​AAG​CGC​TGC​CAG​GGA	EMSA, *rpoS* amplification
P*rpoS*FTRv	AAA​AAA​AAC​CCC​CCC​CCT​TTA​CTG​AGA​GCC​ATT​G	EMSA, *rpoS* amplification
PT7*rsmY*Fw	TTG​CGG​CCG​CTT​TTT​T**TAA​TAC​GAC​TCA​CTA​TAG​G**GTT​CTA​AGA​TTG​GAT​CCA​CTG	EMSA, *rsmY* amplification
P*rsmY*FTRv	AAA​AGC​GGC​CGC​AAA​AAA​AAC​CCC​CCC​CCG​CCG​AAG​CGG​GGT​TTT​CCA​G	EMSA, *rsmY* amplification

^a^ Pseudomonas Reference Culture Collection (http://artemisa.eez.csic.es/prcc/).

^b^ Restriction sites are underlined, inserted T7 polymerase promoter is indicated in bold and sequences used to hybridize with ATTO700-labelled DNA oligonucleotide are highlighted in gray.

### DNA Techniques

Digestion with restriction enzymes, dephosphorylation, ligation and electrophoresis were carried out using standard methods (Ausubel et al., 1987; Sambrook and Russell, 2001) and following manufacturers’ instructions. Plasmid DNA isolation and recovery of DNA fragments from agarose gels were carried out using QIAGEN miniprep and gel extraction kits, respectively. Competent cells were prepared using calcium chloride, and transformations were performed using standard protocols (Sambrook and Russell, 2001). Electrotransformation of freshly plated *Pseudomonas* cells was performed as previously described (Choi et al., 2006).

### Triparental Conjugations

Transfer of plasmids from *E. coli* to *P. putida* strains was performed by triparental matings using as a helper for RP4 transfer functions *E. coli* (pRK600) or SM10 *λpir* (pUX-BF13), the latter when miniTn7 transposase was required for intergenic site-specific insertion of miniTn7 derivatives near *glmS* (Koch et al., 2001), used to tag the wild type and each *rsm* mutant (Table 1) for rhizosphere assays (see below). For each strain, cells were collected from 0.5 ml of overnight LB cultures via centrifugation, then washed and suspended in 50 µL of fresh LB, and finally spotted on nitrocellulose filters (0.22 µm pore diameter) on LB-agar plates. After overnight incubation at 30°C, cells were scraped off from the mating filter and suspended in 2 ml of M9, and serial dilutions were plated on selective medium (M9 with citrate and the appropriate antibiotics) to select exconjugants and counter-select donor, helper, and recipient strains.

### Purification of Total RNA and RNA from Rsm-RNA Complexes

Previously constructed derivatives of expression vector pME6032 harboring each *rsm* gene (Huertas-Rosales et al., 2016) were used to express His-tagged Rsm proteins in *P. putida* KT2440. Overnight cultures (10 ml) of wild type KT2440 harboring each construct were inoculated in 500 ml of LB medium. Three biological replicates were run in parallel. Cultures were incubated at 30°C under shaking until an OD_660_ of 0.8 was reached. At that point, expression of each His-tagged-Rsm protein was induced by the addition of IPTG to a final concentration of 0.5 mM. Cultures were allowed to grow for six additional hours. Aliquots of 1.5 ml were then harvested by centrifugation, instantly frozen with liquid nitrogen and stored at −80°C for total RNA purification. Cells from the remaining culture volume were also harvested and pellets were stored at −80°C until use. His-tagged Rsm-RNA complexes were isolated using Ni-NTA Fast Start purification kit (Qiagen). Three replicate extractions were done for each culture replica. Elution aliquots were analyzed by SDS-PAGE.

Total RNA and RNA from Rsm-RNA complex was extracted using RNA isolation kit (Macherey-Nagel) following the manufacturer’s instructions. RNA samples were subsequently treated with RNase-free DNase I (Turbo DNA-free kit, Ambion) to remove DNA traces, as specified by the supplier. Total RNA quality was assessed using Agilent RNA 6000 Nano Kit (Agilent Technologies) in the Agilent 2100 Bioanalyzer. RNA concentration was measured using Qubit RNA BR assay kit (Life Technologies). 1 µg of RNA was used for rRNA depletion using Ribo-Zero rRNA Removal Kit (Illumina). One of the biological replicates of RsmA and one of RsmI did not meet the required quality and quantity standards and were not used in further analysis.

### Generation of c-DNA Libraries and Sequencing

The generation of cDNA libraries was carried out using NEBNext Ultra Directional RNA Library Prep kit for Illumina (NEB). Dual Index Primers Set one was used to generate bar-coded multiplex libraries (NEB). Library QC was performed using bioanalyser HS kit (Agilent biotechnologies). cDNA libraries were quantified using qPCR (Kapa Biosystems). Libraries were pooled at the desired concentrations, denatured and loaded for sequencing according to manufacturer’s instructions. Sequencing was performed on the Illumina MiSeq Benchtop Sequencer to generate 2 × 75 bp reads. The number of reads obtained ensured a minimum of 76× coverage of the whole genome for control RNA and a minimum of 60× coverage for Rsm-bound RNA.

### Bioinformatic Analysis

Filtered reads were aligned to reference genome *P. putida* KT2440 (GenBank; RefSeq NC_002947.3) with Bowtie v2 (Langmead et al., 2009). Alignment. sam file was analyzed using MACS v14 to identify and evaluate the significance of reads-enriched regions in the genome, the output being one file containing the peak chromosome coordinates, and one containing the genome coordinates, summit, *p*-value, fold_enrichment and false discovery rate (FDR) of each peak (Zhang et al., 2008). The average number of tags in the control samples after filtering was approximately 2,220,000 (RsmA), 1,420,000 (RsmE), and 1,654,000 (RsmI); the average number of tags in the Rsm-bound samples after filtering was approximately 1,302,000 (RsmA), 550,000 (RsmE), and 1,054,000 (RsmI). Only those peaks present in the three technical replicates were considered. Identity and annotation of the targets above the cut-off values were further validated by individually inspecting the corresponding chromosomal regions in the *Pseudomonas* genome database (www.pseudomonas.com; Winsor et al., 2016).

### EMSA Analysis of *in vitro* RNA-Protein Binding

For purification of Rsm proteins, overnight cultures (10 ml) of *P. putida* KT2440 harboring plasmids pME6032-*rsmA*, pME6032-*rsmE*, and pME6032-*rsmI* (Huertas-Rosales et al., 2016) were used to inoculate 490 ml fresh LB medium supplied with Tc. Cultures were grown at 30°C and 200 rpm until reaching an OD_660_ of 0.8. At this point, IPTG (0.5 mM) was added to induce the expression of the proteins. After 6 h of further growth, cells were harvested by centrifugation and pellets subsequently stored at −80°C. Protein purification was carried out using QIAexpress Ni-NTA Fast Start Kit (Qiagen), following the manufacturers’ instructions.

Electrophoretic mobility shift assays (EMSA) were carried out following a method described previously (Romero et al., 2018). DNA templates corresponding to the target gene sequences were amplified by PCR using primers that incorporated a T7 promoter at the 5′ end and a 17-nt extension at the 3′ end (Table 1). The purified PCR product was used for RNA synthesis *in vitro* using the MAXIscript T7 kit (Life Technologies). The RNA obtained was visualized by hybridization of an ATTO700-labeled DNA primer to the 3′ extension of the RNA. Rsm proteins were incubated with target gene RNA (5 or 10 nM) in 1 × binding buffer [25]. Binding in the absence or presence of unlabeled competitor RNA (100-fold excess) was carried out for 30 min at 30°C. Then Bromophenol Blue was added (0.01%, wt/vol) before electrophoresis on 6% (w/v) non-denaturing polyacrylamide TBE gel at 4°C. Imaging was performed using a 9201 Odyssey Imaging System (LI-COR Biosciences).

**TABLE 2:** Summary of the high throughput data analysis of Rsm targets in *P. putida* KT2440.

Biological replica	total peaks	CI 95^a^ fold enrichment (lower limit)	Cut-off value	CI 95 -10 × log (*P*val) (lower limit)	Cut-off value	peaks above cut-off values	peaks in all biological replicas	unique RNA targets^b^
RsmA1			2.15		130		243	241
RsmA2	595	2.33		135		266		
RsmA3	596	2.33		128		244		
RsmE1	908	4	4	180	170	327	270	261
RsmE2	851	4.22		150		320		
RsmE3	850	4.62		181		402		
RsmI1	557	2.68	2.5	153	145	244	209	206
RsmI2								
RsmI3	596	2.53		137		238		

Shadowed in gray the discarded replicas.

^a^ Confidence intervals of each data distribution, *α* = 0.05.

^b^ After discarding redundancies where the analysis identifies more than one peak in a single RNA molecule.

### Growth with Different Carbon and Nitrogen Sources

BIOLOG plates (Biolog Inc. Hayward, CA, USA) were used for initial assessment of growth of KT2440 and the triple ∆*rsmIEA* mutant with different nitrogen and carbon sources. Cultures grown overnight at 30°C in M9 minimal medium with glucose as carbon source were used for inoculation in the plates at an initial OD_660_ of 0.05. Turbidity was measured at different times over 24 h in a Tecan Sunrise microplate reader. Further experiments were done in 96-well plates using M9 or M8 with the indicated carbon or nitrogen sources, at a final concentration of 5 mM. Growth was followed for 24 h at 3°C with continuous shaking in a Bioscreen apparatus C MBR equipped with a wide band filter (420–580 nm).

### Competitive Root Colonization Assays

Surface sterilization, germination of corn seeds, and bacterial inoculation of the seedlings were performed as described previously (Ramos-González et al., 2013). Briefly, at least six two-days old seedlings were inoculated with a 1:1 mix (∼5 × 10^5^ CFU/strain) of KT2440Tn7-Km, as the wild type, and the wild type or mutant derivatives tagged with miniTn7Sm by triparental conjugation as described above. Inocula sizes were monitored by plating on LB-agar supplied with kanamycin or streptomycin. After 7 days, bacteria were recovered from the rhizosphere or the root tip as specified (Ramos-González et al., 2013) and the number of cells of each strain in the population was estimated by plating on LB-agar supplied with kanamycin or streptomycin. Data are presented as the index of colonization fitness (Ramos-González et al., 2013). SigmaStat software package (Systat software) was used for statistical analysis. The data were compared using Student’s *t*-test for independent samples (*p* < 0.05).

### Data-Availability

Raw and processed data files have been deposited in the Gene Expression Omnibus Database (www.ncbi.nlm.nih.gov/geo/) and are available under accession number GSE154204.

## Results and Discussion

### Identification of RNAs Bound to Rsm Proteins

Affinity purification of RNA-protein complexes followed by sequencing analysis (RAP-Seq), was carried out to identify genes that could potentially be regulated at the post-transcriptional level by RsmA, RsmE or RsmI in *P. putida* KT2440. The methodology was similar to that previously described for genome-wide mapping of targets for RsmN in *Pseudomonas aeruginosa* (Romero et al., 2018) and is summarized in Supplementary Figure S1. Each recombinant His-tagged Rsm protein (RsmA-His_6_, RsmE-His_6_ and RsmI-His_6_, previously described; Huertas-Rosales et al., 2016) was over-expressed in KT2440 as described in Materials and Methods. Proteins were purified by affinity chromatography and their associated target RNAs subsequently isolated. As control for transcription levels, total RNA was isolated from each culture in parallel. Total and Rsm-bound RNA were analyzed for purity, quantified and converted to cDNA for Illumina sequencing. One of the three biological replicates of RsmA and one of RsmI were below optimal quality and quantity and were not used in further analysis. Sequence reads from the cDNA libraries were mapped to the genomic sequence of *P. putida* KT2440 and analyzed to identify the regions corresponding to transcripts that were significantly enriched in the Rsm-bound RNA population with respect to the total RNA controls. Rsm-enriched RNAs that were not represented in the three technical replicates from each culture were not included in further analysis.

**FIGURE 1:**
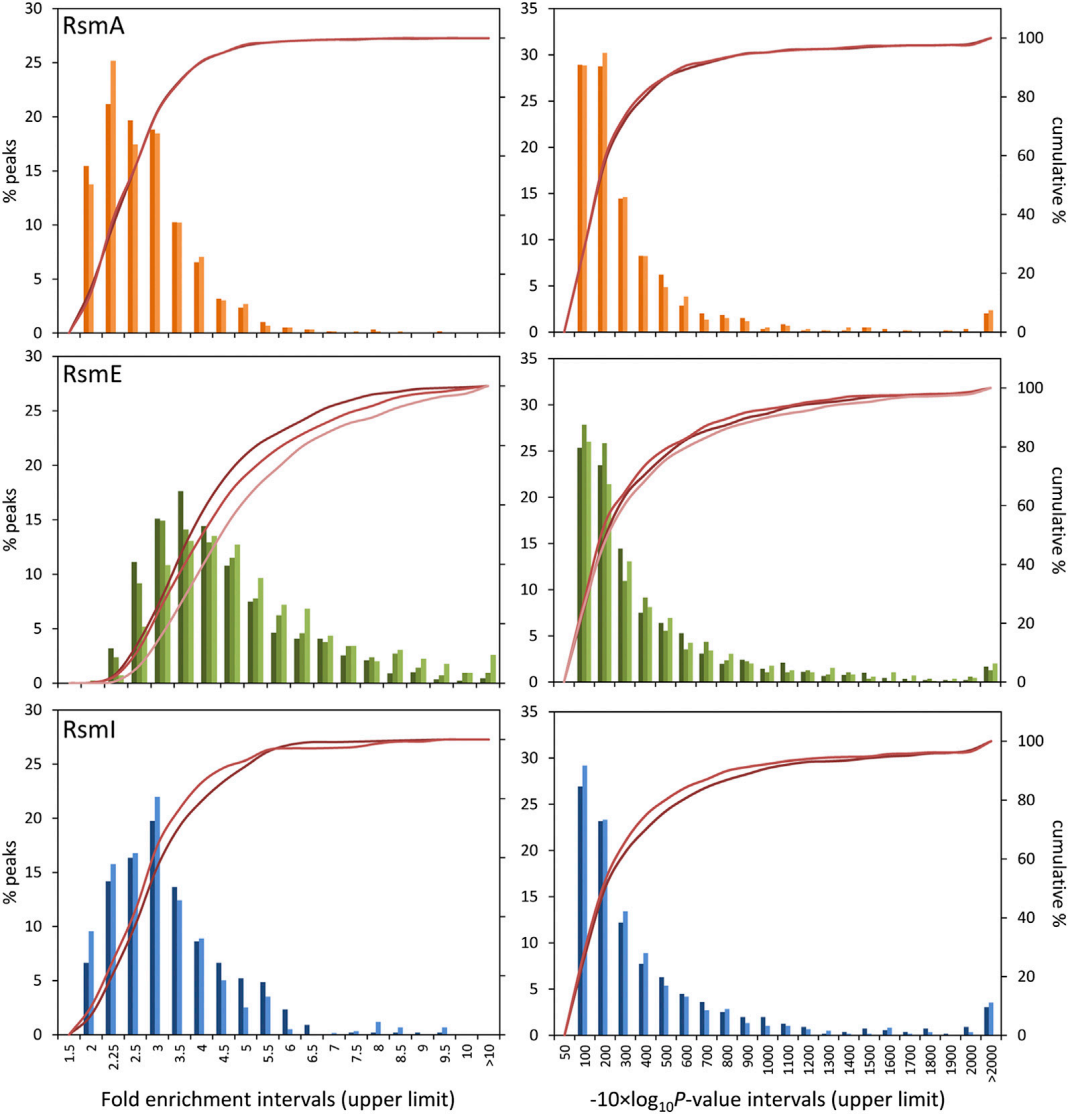
Distribution of fold enrichment **(left)** and −10 × Log_10_(*p*-value) scores **(right)** data of overrepresented RNA sequences associated to each Rsm protein (orange, RsmA; green, RsmE; blue, RsmI) with respect to total RNA. Data were grouped in the value intervals indicated in the *X*-axis, where the number corresponds to the upper limit of each interval. The histograms (primary *Y*-axis) show the frequency of targets in each interval. The crimson lines (secondary *Y*-axis) correspond to the cumulative percent values. The different color intensities in the histograms and lines correspond to the different biological replicates for each protein.

A first noticeable result was that the number of sequences corresponding to RsmE-bound transcripts overrepresented with respect to total RNA was much higher than those associated to RsmA or RsmI (Table 2). Data were grouped in intervals and histograms were built to analyze the distribution of fold-enrichment (FE) values and *p*-value (PV) data–shown as -10×log_10_(*p*-value) for ease of representation–in each case (Figure 1). In all cases, the distribution was similar between biological replicates for each Rsm regulator. The distribution of FE values was similar for RsmA and RsmI, with slightly lower values in the former (Table 2). For RsmE, the distribution was different and the average values higher. These different values between Rsm proteins could reflect differences in expression of each construct. In fact, controls for each protein indicate that higher amount of RsmE than of the other two proteins is recovered after purification (Supplementary Figure S2).

**TABLE 3:** Annotation and functional classification of common targets for the three Rsm proteins in *P. putida* KT2440.

Locus	Gene	Annotation	Notes
Metabolism			
PP_0053		sulfide:quinone oxidoreductase	Sulfur metabolism
PP_0158	*gcdH*	Glutaryl-CoA dehydrogenase	Operon (PP_0159: family III CoA-transferase)
PP_0292	*hisA*	Phosphoribosylformimino-5-aminoimidazole carboxamide ribonucleotide isomerase	Operon *hisBHAF*; histidine synthesis
PP_0626	*ndh*	NADH dehydrogenase	
PP_0711	*ycaC-I*	Putative hydrolase	Isochorismatase family
PP_1032	*guaA*	Glutamine-hydrolyzing GMP synthase	Purine metabolism
PP_1073	*glpD*	Aerobic glycerol-3-phosphate dehydrogenase	
PP_2080	*gdhB*	NAD-specific glutamate dehydrogenase	
PP_2217		Enoyl-CoA hydratase	
PP_2437		Acyl-CoA dehydrogenase	
PP_2640		GNAT family acetyltransferase	
PP_2681	*pqqD-II*	Pyrroloquinoline quinone biosynthesis chaperone	
PP_4571	*cysK*	Cysteine synthase K	Cysteine biosynthesis
PP_5003	*phaA*	poly (3-hydroxyalkanoate) polymerase	Synthesis of carbon/energy reserve polymers
PP_5079	*aroK*	Shikimate kinase	Biosynthesis of aromatic amino acids
PP_5199	*ubiH*	2-Octaprenyl-6-methoxyphenyl hydroxylase	Ubiquinone biosynthesis
Protein synthesis, degradation and modification			
PP_1434	*era*	GTPase Era	Maturation of 16S rRNA and assembly of the 30S ribosomal subunit
PP_1443	*lon-I*	DNA-binding ATP-dependent protease	Might indirectly regulate the levels and activity of sRNAs through stability of hfq
PP_3620	*ycaO*	Ribosomal protein S12 methylthiotransferase accessory factor	Post-translational peptide modification
PP_4559	*def-II*	Peptide deformylase	Processing of nascent peptides
PP_5364	*clsA*	Cardiolipin synthase	
Transport and secretion			
PP_0907		RND family multidrug transporter	Antimicrobial resistance
PP_1015	*gtsA*	Mannose/glucose ABC transporter substrate-binding protein	
PP_2195		Periplasmic putrescine-binding protein	
PP_3089	*hcp1*	Type VI secretion system effector protein	Part of the K1-T6SS
PP_3099	*tssC1*	Type VI secretion system	Part of the K1-T6SS
PP_3108	*tke2*	Type VI secretion system effector protein	Part of the K1-T6SS
PP_4542		ABC transporter ATP-binding protein/permease	
Stress response			
PP_1210	*dps*	DNA-binding stress protein	
PP_3156		Universal stress protein family	
PP_3234		HSP20 family heat shock protein	Putative chaperone
PP_3312		HSP20 family heat shock protein	Putative chaperone
PP_4541	*ppiA*	Peptidyl-prolyl *cis*-trans isomerase A	Protein folding; stress response and biofilm in different bacteria
Signal transduction and regulation			
PP_0173		Transcriptional factor-like protein	Winged helix DNA binding domain
PP_0546		Sigma-54 dependent transcriptional regulator	Putative acetoin metabolism regulator
PP_0563		Two-component system respose regulator - GGDEF domain	c-di-GMP turnover
PP_1492	*wspE*	Two-component system sensor histidine kinase/response regulator (CheA/WspE)	Part of the *wsp* cluster (biofilm formation)
PP_3761	*cfcA*	Two-component system sensor histidine kinase/response regulator	CfcR phosphorylation and activation
PP_3765	*turB*	H-NS family protein (MvaT homolog)	Repressor of gene expression
PP_3832	*rsmE*	Post-transcriptional regulatory protein RsmE	
PP_4099	*uvrY*	Two-componenent system response regulator	Operon with *uvrC* (nucleotide excision repair)
Non-coding RNAs			
PP_mr05	*rsmY*	Non-coding RNA	
PP_mr44		Non-coding RNA	
PP_mr52		Non-coding RNA	
DNA recombination and transposition			
PP_1813		comEA-like protein	DNA binding and recombination domain
PP_1865		Transposase ISPpu8 + intergenic region	
PP_2114		Transposase ISPpu8 + intergenic region	
intergenic		Putative ISPpu8 insertion site	Long palindromic region downstream PP_3547
PP_4318		Transposase ISPpu8 + intergenic region	
Cell envelope and appendages (LPS, EPS, pili)			
PP_0063	(*lpxL*)	Lipid a biosynthesis lauroyl acyltransferase	LPS synthesis
PP_2926	*udg*	UDP-glucose 6-dehydrogenase	may be involved in LPS synthesis
PP_3139		Group 1 family glycosyl transferase	Part of the *pea* operon - EPS synthesis
PP_4795		Lipoprotein	LptE family - LPS assembly
PP_4920		Lipoprotein	PdaC superfamily (polysaccharide deacetylase)
PP_5083	*pilM*	Type IV pili biogenesis protein PilM	Operon 5083-5079 (type IV pili + *aroK*)
Hypothetical/unknown function			
PP_5720		Pseudogene	
PP_0085		Hypothetical protein	YqjD/ElaB family (stress response?)
PP_1499		Hypothetical protein	
PP_1887		Hypothetical protein - YD repeat domain	Repeats may bind carbohydrates
PP_2219		Hypothetical protein	
PP_2345		Hypothetical protein	
PP_2396		Hypothetical protein	Periplasmic protein
PP_3007		Hypothetical protein	
PP_3010		Hypothetical protein	Putative lipoprotein
PP_3130		Hypothetical protein	Glycoside hydrolase family (EPS turnover?)
PP_3580		Hypothetical protein	
PP_3662		Hypothetical protein	Nucleotide 5′-monophosphate nucleosidase YgdH-like superfamily
PP_3901		Hypothetical protein	Predicted phage protein
PP_3909		Hypothetical protein	
PP_3963		Hypothetical protein	Stress-induced protein (KGG repeat) domain
PP_4793		Hypothetical protein	
PP_5099		Hypothetical protein	
PP_5209		Hypothetical protein	FliL-like protein
PP_5232		Hypothetical protein	
PP_5395		Hypothetical protein	Branched-chain polyamine synthase domain

The analysis of distributions and the confidence intervals calculated for each technical replicate were the basis to establish cut-off values for further analysis of Rsm targets (Table 3). Is should be noted that in this analysis we opted for rather strict parameters in order to take into account sequences strongly overrepresented in the Rsm-bound RNA population with respect to the total RNA controls, and also to minimize the number of potential false positives, at the expense of missing some RNA sequences that are actual targets of these proteins. Thus, the following cut-off values were established: PV > 130, FE ≥ 2.15 for RsmA; PV > 170, FE ≥ 4 for RsmE; PV > 145, FE ≥ 2.5 for RsmI. Targets for which either value was below the cut-off in one of the replicates were discarded.

**FIGURE 2:**
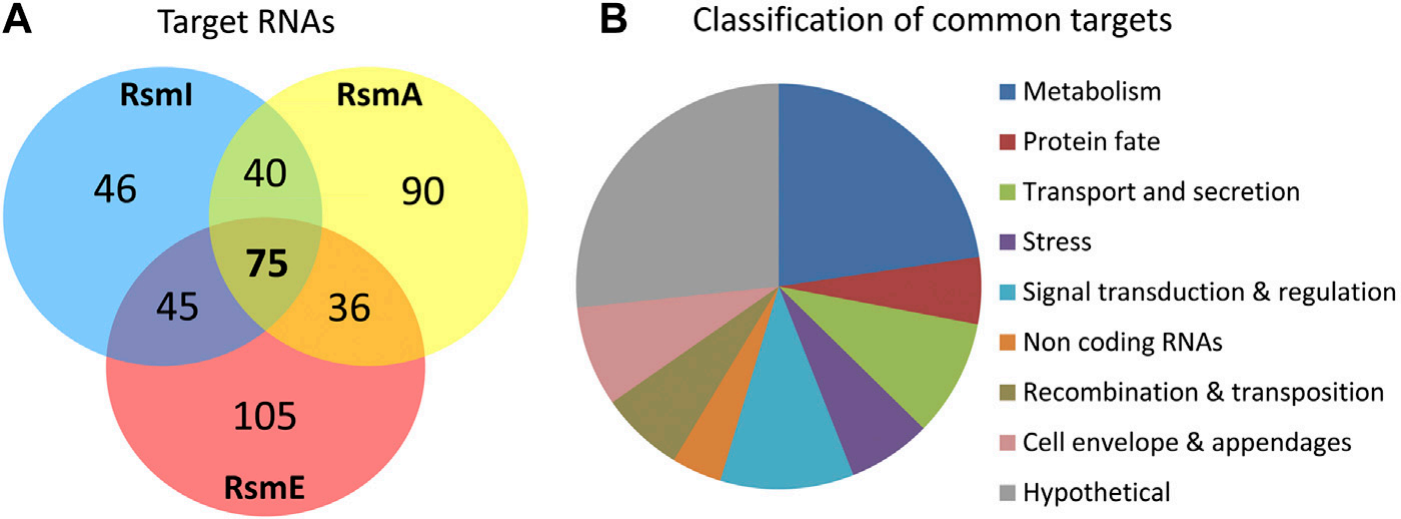
**(A)** Venn diagram summarizing the number of targets identified for the three Rsm proteins in *P. putida* KT2440, according to the selection parameters detailed in the text. **(B)** Graphical representation of the different functional categories encompassing the 75 common targets.

Using these parameters, 241, 261, and 206 RNA sequences were identified as targets for RsmA, RsmE and RsmI, respectively (Supplementary Table S1), corresponding to 437 unique transcripts, with 75 targets being shared by all three Rsm proteins and between 36 and 45 shared by two of them (Figure 2A). Interestingly, around 40% of the RsmE and RsmA targets are exclusive for each protein, while only 22% of the RsmI targets are unique for this paralog. It should be noted that the cDNA libraries generated in this study were not strand-specific and therefore, did not allow distinguishing the DNA strand to which the transcript corresponds. Consequently, in some cases where divergently transcribed genes are adjacent in the genome, it is not straightforward to discern which of them is the actual target, although the length of overlap between the enriched sequences and each gene, and the location of the summit (i.e. the position of maximal overlap of the reads corresponding to one region) can indicate the most likely target. Despite this limitation, the above results indicate that at least 12% of the transcripts in *P. putida* KT2440 are bound *in vivo* by Rsm proteins under the conditions tested in this study. The data provide a broad overview of the regulon and potential functions of these riboregulators.

### Analysis of RsmA, RsmE and RsmI Targets

**FIGURE 3:**
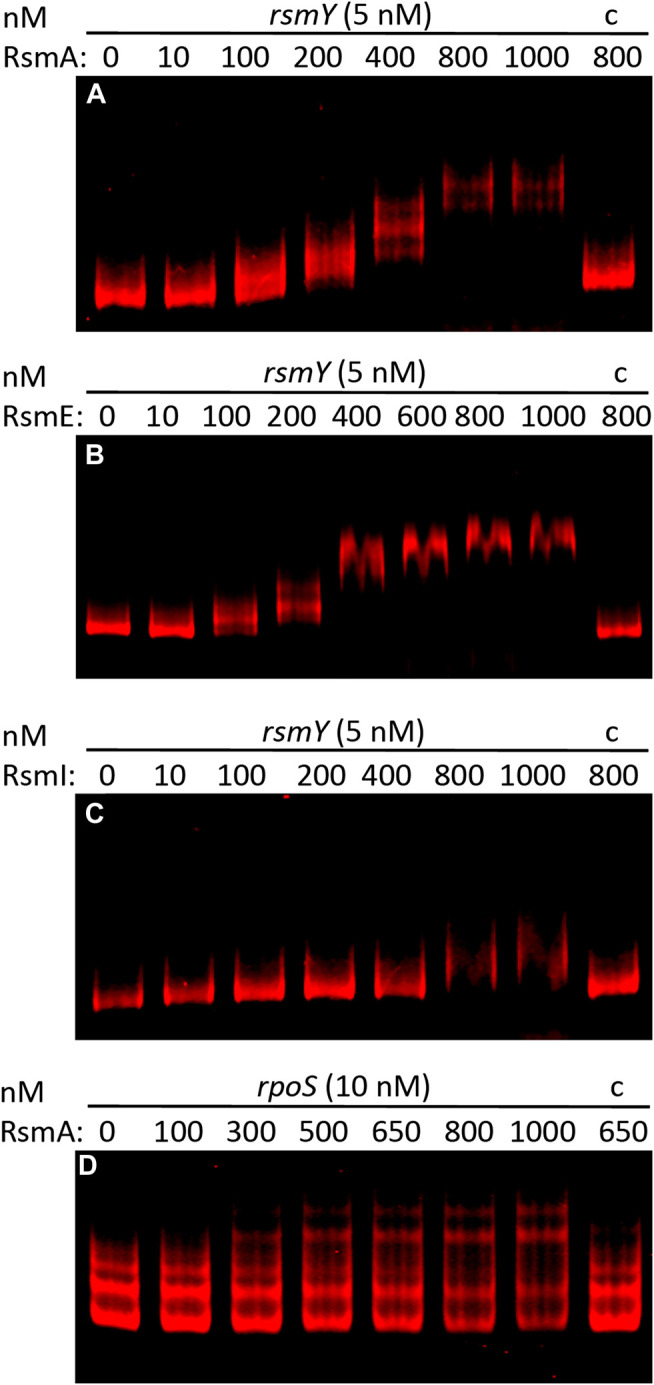
Binding of RsmA-His_6_ **(A)**, RsmE-His_6_ **(B)** and RsmI-His_6_ **(C)** to full-length *rsmY* RNA transcribed *in vitro*, and of RsmA-His_6_ to a synthetic RNA fragment comprising the untranslated upstream region of *rpoS* **(D)**. EMSAs were carried out using fluorescently labeled RNA (5 or 10 nM) and incubated in the absence or presence of increasing concentrations of purified Rsm proteins, as indicate above each lane, or in the presence of 100-fold excess of unlabelled RNA competitor and the indicated concentration of protein **(C)**.

The 75 targets common to the three Rsm proteins are compiled in Table 3, broadly classified according to their functions. As reflected in Figure 2B, about half of the common targets correspond to two categories: metabolism-related and hypothetical proteins. The fact that functions related to central metabolism and carbon storage are among these common targets is not unexpected, since carbon metabolism was at the origins of the identification of the CsrA/Rsm family of proteins (Sabnis et al., 1995). Other expected elements include RNAs corresponding to Rsm proteins themselves: *rsmE* is a target for the three proteins, and *rsmA* is recognized by RsmE, confirming previous expression data that indicated the existence of self- and cross-regulation of these proteins (Huertas-Rosales et al., 2016). Also expected was the small non-coding RNA *rsmY*, known to bind and titrate Rsm proteins (Sonnleitner and Haas, 2011; Janssen et al., 2018). Binding of *rsmY* to the three Rsm proteins could be confirmed *in vitro* by EMSA analysis (Figure 3), serving as positive control that the high throughput methodology used was successful, although the affinity appears to be different in each case, being RsmI the protein that required higher concentrations for binding to be detected. A second small RNA previously identified in *P. putida*, *rsmZ* (Huertas-Rosales et al., 2016), involved in titration of Rsm proteins in other bacteria (Janssen et al., 2018), is among the targets common to RsmA and RsmE (Table 4, Supplementary Table S1). Other non-coding RNAs could also be identified as bound to one, two or the three Rsm proteins (Table 2). This might suggest a possible ancillary role in titration of Rsm proteins, which led us to analyze them in some detail. Secondary structure predictions indicate that GGA motifs in short stem-loop structure, typical targets for CsrA/RsmA recognition (Dubey et al., 2005; Duss et al., 2014), are present in at least some of these RNA molecules, namely PP_mr15, PP_mr49 and PP_mr55 (Supplementary Figure S3). However, while *rsmY* and *rsmZ* show several of these motifs, only one per molecule was present in the other three. Also, conserved GacA binding sites, involved in transcriptional regulation of Rsm-titrating RNAs in other *Pseudomonas* species (Brencic et al., 2009) could be identified in the regions upstream *rsmY* and *rsmZ*, being only partially conserved in PP_mr55 (Supplementary Figure S4), and absent in the remaining RNAs (not shown). All these data suggest that *rsmY* and *rsmZ* are the main, if not the only, true antagonists of Rsm proteins in *P. putida*. The remaining non-coding RNAs that are targets of these proteins may regulate further downstream elements in the Gac/Rsm cascade.

**TABLE 4:** Non-coding RNAs identified as targets for each Rsm protein.

		Fold-enrichment^a^		
Locus^b^	Length (bp)	RsmA	RsmE	RsmI
PP_mr05 (*rsmY*)	127	8.36	16.46	6.88
PP_mr15	209	3.83	5.37	(3.49)
PP_mr22 (*rsmZ*)	134	4.58	11.58	(5.11)
PP_mr44	385	2.87	6.49	3.22
PP_mr49	97	3	—	(3)
PP_mr52	149	2.36	6.96	4.72
PP_mr53	395	3.93	(3.76)	3.53
PP_mr55	82	—	5.20	—
PP_mr57	254	8.17	—	—
PP_mr59	97	2.42	(2.96)	(2.1)

^a^Values in parentheses indicate targets below the *p*-value and/or fold-enrichment cutoffs. Minus sign indicates the target is not present in one or more of the enriched RNA replicas.

^b^ Annotated according to 54.

Additionally, several intergenic sequences were found among the targets for the three proteins. These correspond to regions adjacent to the different copies of the *IS*Ppu8 transposase in the genome of *P. putida* KT2440 or are potential insertion sites (or remnants of previous insertions) of this transposase, based on their sequence identity with *IS*Ppu8 flanking regions. Whether Rsm proteins influence the activity of this mobile genetic element in KT2440 is an interesting issue that deserves further study.

As mentioned above, a significant number of targets seem to be exclusive for one of the three proteins. A few of these correspond to enriched peaks that were below the established cut-off parameters in some samples, and therefore could represent common targets showing different affinities for each protein, with a strong preference for one of them. Such is the case, for example, of PP_0013 (*gyrB*), which is among the above-cut-off targets for RsmA but slightly below the FE and/or *p*-value cut-off for the other two proteins. Other targets are only enriched in association with one of the Rsm proteins and are not present in the RNA population associated to either of the other two, indicating they are truly specific for that Rsm homolog, e.g. PP_1656 (*relA*) for RsmA, PP_0168 (*lapA*) for RsmE, or PP_1803 (*wpbV*) for RsmI. Identifying the molecular basis for such specificity will require detailed bioinformatics analysis combined with *in vitro* and *in vivo* assays.

Supplementary Figure S5 provides data corresponding to β-galactosidase activity of several translational fusions of identified targets to *lacZ* in the wild type KT2440 and a triple *rsmArsmErsmI* deletion mutant, ΔIEA (Huertas-Rosales et al., 2016). In most cases, expression was enhanced in the mutant, indicating that the observed binding to their RNA targets leads to translation repression by these proteins; these results are consistent with previous observations on their influence upon biofilm-related elements (see below). However, the inverse was true for two of the tested fusions, those corresponding to PP_1088 (*argG*, involved in arginine synthesis; Ramos-González et al., 2016) and PP_4482 (part of the gene cluster encoding the main arginine transporter and its regulator; Barrientos-Moreno et al., 2020). In these cases, expression was approximately halved in the mutant with respect to the wild type.

### c-Di-GMP Signaling and Biofilm-Related Targets

As indicated in the Introduction, the three Rsm proteins have been shown to bind a specific site in the mRNA corresponding to the response regulator with diguanylate cyclase activity CfcR, and a triple *rsm* mutant in KT2440 shows increased levels of c-di-GMP and altered biofilm dynamics (Huertas-Rosales et al., 2016; Huertas-Rosales et al., 2017). Intriguingly, *cfcR* mRNA was not among the common targets listed in Table 3, being only found as target for RsmE and RsmI. The *cfcR* transcript is actually among the RsmA-bound RNA sequences overrepresented with respect to the control RNA, but the FE values (1.82 and 1.88 in each replicate, respectively) are below the established cut-off. This could indicate that binding of RsmA to the mRNA of *cfcR in vivo* is hampered by competition with the other two Rsm proteins, a possibility that has been previously suggested, based on *in vivo* expression data of *cfcR* compared to the high affinity observed for the RsmA/*cfcR* mRNA interaction *in vitro* (Huertas-Rosales et al., 2017).

Besides *cfcR*, transcripts from four other genes encoding proteins predicted to participate in c-di-GMP turnover and signaling were identified in this analysis, one of them (encoded by locus PP_0563) as target of the three proteins, and the rest (PP_0386, PP_0914, PP_2505), as targets of RsmE. Of these, the proteins encoded by PP_0563 and PP_2505 present GGDEF domains, characteristic of diguanylate cyclases. The first corresponds to GcbA, a diguanylate cyclase conserved in *Pseudomonas*, which has been reported to influence initial attachment and swimming motility (Petrova et al., 2014; Xiao et al., 2016). PP_0914 corresponds to the EAL domain-containing phosphodiesterase BifA, described to regulate biofilm development in *P. putida* (Jiménez-Fernández et al., 2015), and PP_0386 encodes a protein containing both GGDEF and EAL domains. Other relevant biofilm-related transcripts bound by RsmE included: *lapA*, encoding the main adhesin of *P. putida*, essential for initial attachment and biofilm formation (Hinsa et al., 2003; Martínez-Gil et al., 2010); the first gene in the cellulose synthesis operon; and genes in the operon encoding the species-specific EPS Pea (Nilsson et al., 2011), which is also a target for RsmA. Neither *lapF*, encoding the second relevant adhesin present in KT2440 (Martínez-Gil et al., 2010), nor the other two EPS operons described in this strain (Nilsson et al., 2011) were identified in this analysis.

**FIGURE 4:**
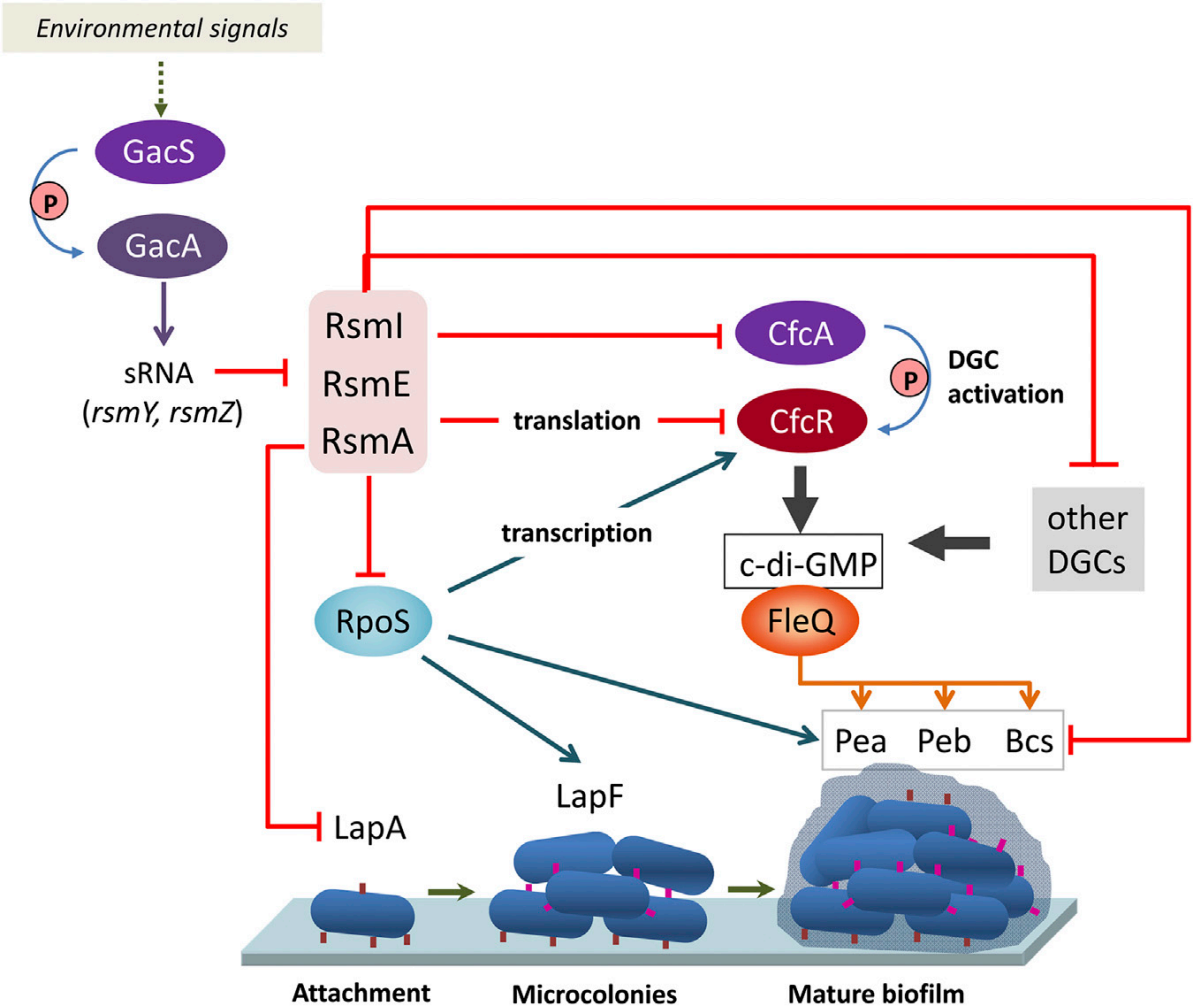
Schematic view of the connection between the Gac/Rsm signaling cascade with biofilm formation through c-di-GMP turnover, adhesins and exopolysaccharides. The Rsm proteins exert a complex regulation on the response regulator with DGC activity CfcR, at the transcriptional (via RpoS), translational (via direct binding), and post-translational (via the histidine kinase CfcA, required for phosphorylation of CfcR) levels. Direct influence of Rsm proteins on structural elements of the biofilm matrix, and indirect influence via RpoS and the c-di-GMP associated regulator FleQ are also depicted. Blue arrows indicate positive regulation and red lines negative regulation.

Since altered expression of translational fusions corresponding to some of the genes indicated aboved has been previously observed in a triple *rsmAEI* mutant (Huertas-Rosales et al., 2016), it is likely that the observed expression changes are due to an indirect effect of Rsm proteins through other regulators. One such regulator is the stationary phase sigma factor RpoS, which controls expression of *lapF* (Martínez-Gil et al., 2010). RpoS was found to be negatively regulated by RsmA In *P. protegens* CHA0 (Heeb et al., 2005), and the mRNA corresponding to *rpoS* (PP_1623) is among the targets for RsmA in KT2440 (Supplementary Table S1). Moreover, binding of RsmA to an *in vitro* transcribed RNA fragment containing the ribosome binding site and start codon of *rpoS* was confirmed via EMSA (Figure 3). Given that expression of *cfcR* is also regulated by RpoS (Matilla et al., 2011), these results support the previously proposed model whereby Rsm proteins exert a dual control, direct and indirect, on *cfcR* (Huertas-Rosales et al., 2017). Remarkably, the gene *cfcA*, encoding a sensor histidine kinase essential for activation of CfcR (Ramos-González et al., 2016), is among the targets for the three proteins, indicating that c-di-GMP signaling through CfcR is tightly regulated by the Gac/Rsm network. A schematic view of this signaling cascade connecting external stimuli with biofilm formation through Rsm elements is depicted in Figure 4.

### Influence of Rsm Proteins on Nutrient Utilization and Rhizosphere Fitness of *P. putida* KT2440

**FIGURE 5:**
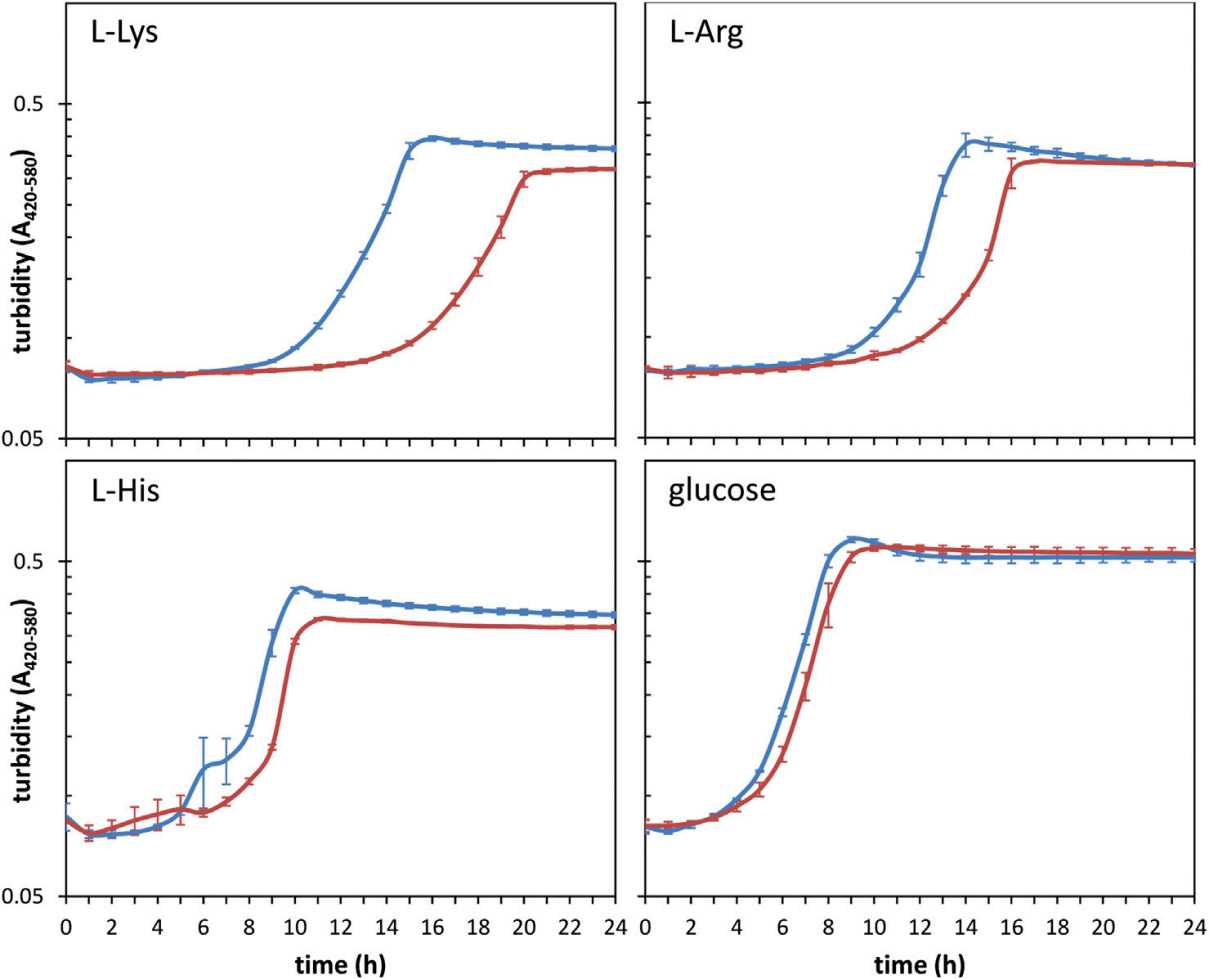
Growth of KT2440 (blue) and the triple *rsm* mutant (red) in M9 minimal medium with 5 mM L-lysine, L-arginine, L-histidine or glucose as carbon source. Cultures were grown in 96-well plates at 30°C and OD_420–580_ was read at 1-h intervals in an automated BioScreen equipped with a wide band filter (420–580 nm) to minimize potential alterations due to changes in medium color. Data are averages and standard deviations of experiments done in duplicate, with three technical replicas each.

Among the shared targets for the three riboregulators, about a third of the transcripts with known or predicted functions correspond to genes with metabolism-related roles in KT2440 (Figure 2B and Table 4). This was expected since previous findings have established direct and indirect connections between the CsrA/Rsm system and metabolism as well as carbon storage functions (Sabnis et al., 1995; Yang et al., 1996; Pannuri et al., 2016; Romero et al., 2018). Furthermore, a significant number of targets identified for one or more of the Rsm proteins included transcripts from genes related to transport of nutrients (Figure 2B and Table 3). All this prompted us to carry out a preliminary high throughput study comparing the growth of KT2440 wild type strain and a triple *rsmAEI* mutant derivative (ΔIEA; Huertas-Rosales et al., 2016) in BIOLOG plates using 192 and 96 compounds as sole carbon or nitrogen source, respectively. A sample of some of the obtained data is shown in Supplementary Figure S5. Growth differences between the two strains were observed for several compounds, particularly certain amino acids and their derivatives. Further detailed evaluation of growth in some of these compounds confirmed the existence of differences between KT2440 and the triple *rsm* mutant. In particular, a prolonged lag phase was observed in the mutant with L-lysine as carbon source, and to a lesser extent with L-arginine, although the final turbidity reached by both strains with this last amino acid was similar (Figure 5). In contrast, growth differences were less evident with the other basic amino acid L-histidine (Figure 5).

These results evidenced that the Rsm system may play a regulatory role in metabolism and/or uptake of nutrients, particularly of basic amino acids, in KT2440. Hence, potential Rsm-enriched targets explaining these divergences were explored. A survey of the identified RNA molecules related to these processes indicated that those corresponding to *arcD* (PP_1002; arginine-ornithine antiporter) and *hisP* (PP_4483; ATP-binding subunit of a histidine/lysine/arginine/ornithine transporter) are targets for RsmE; *artJ* (PP_0282; L-arginine ABC transporter substrate-binding subunit) and *amaD* (PP_3596; D-lysine oxidase) are targets for RsmA, and *amaB* (PP_5258; L-piperidine-6-carboxylate dehydrogenase) is a target for both RsmE and RsmI (Supplementary Table S1). Based on data from the Kyoto Encyclopedia of Genes and Genomes (www.genome.jp/kegg/), AmaB is involved in catabolic pathways for L-lysine and L-arginine in *P. putida*, participating in the conversion of aminobutanal, N4-acetyl-aminobutanal, and 4-trimethyl-ammoniobutanal into their corresponding butanoate derivatives.

Besides glucose metabolism, a connection has been established in *E. coli* between CsrA and the stringent response (Edwards et al., 2011), a regulatory network triggered by amino acid limitation and controlled by the RelA and SpoT proteins. It should be noted that besides the targets indicated above in relation with arginine and lysine utilization, the *relA* (PP_1656) mRNA is also a target for RsmA (Supplementary Table S1).

**FIGURE 6:**
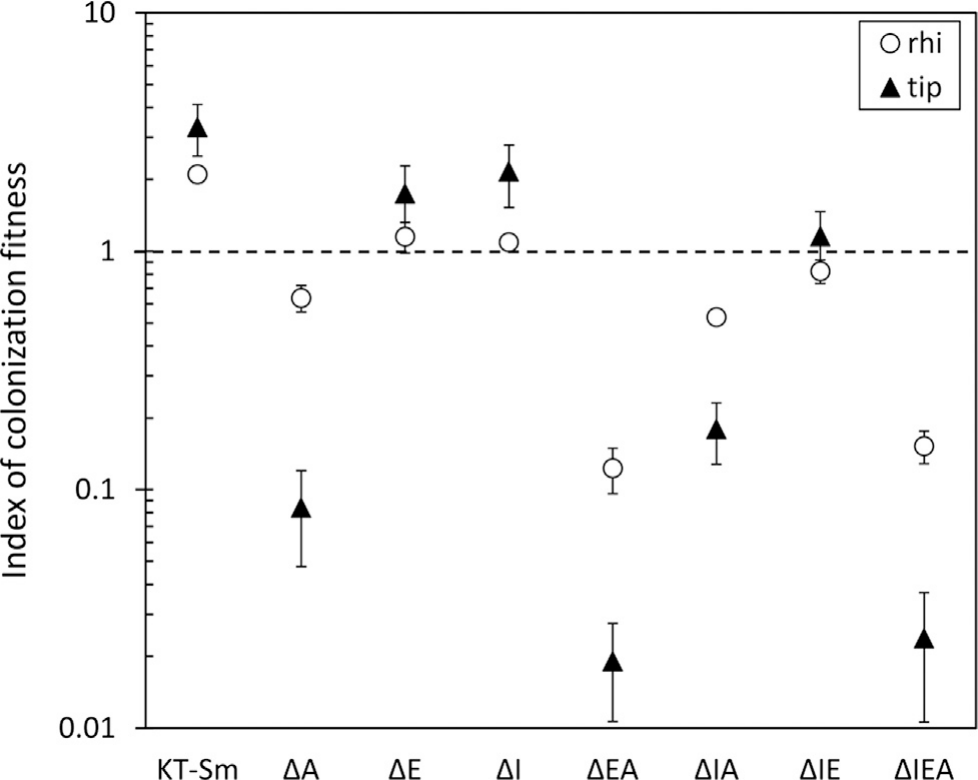
Colonization capacity of mutants in *rsm* genes in competition with the wild type in the rhizosphere (circles) and the root tip (triangles) of corn plants. KT2440 tagged with miniTn7-Km was co-inoculated in a 1:1 proportion with each of the mutants, which were tagged with miniTn7-Sm. To check the potential effect of the antibiotic resistance markers, a competitive colonization assay co-inoculating KT2440 miniTn7-Km and KT2440 miniTn7-Sm (KT-Sm) was also included as control. The index of colonization fitness was measured as 1/[(percentage of recovered wild type vs. each mutant per plant 7 days post-inoculation)/(percentage wt vs. mutant in the initial inocula)]. A value of 1 (broken line) indicates equal colonization capacity. Data are the averages and standard errors for six plants.

Previous reports have established that *hisP* and genes involved in lysine catabolism are preferentially expressed in KT2440 in the rhizosphere of corn plants (Espinosa-Urgel and Ramos, 2001; Matilla et al., 2007). In addition, a connection beween arginine transport and c-di-GMP signaling in this strain has been recently reported (Barrientos-Moreno et al., 2020). These facts, and the influence of Rsm proteins on biofilm formation and surface motility, along with the identification of c-di-GMP turnover elements and other biofilm-related genes as targets of these proteins, made us analyze if mutants in *rsm* genes showed altered fitness in the rhizosphere. Competitive root colonization assays were done in corn (*Zea mays* L.) plants with the wild type and single (ΔI, ΔE, ΔA), double (ΔIE, ΔIA, ΔEA) and the triple (ΔIEA) *rsm* mutants (Huertas-Rosales et al., 2016). For that purpose, germinated corn seeds were inoculated with miniTn7Km-tagged KT2440 and each mutant tagged with miniTn7Sm, in a 1:1 proportion. Plants were sown in sterile sand and the population of each strain was evaluated in the whole root and in the root tip after 7 days. Results are shown in Figure 6. The mutation in *rsmA* caused a slight reduction in competitive colonization of the whole root and a much larger effect when root tip colonization was evaluated. This phenotype was not observed in the other single mutants, nor in the ΔIE double mutant. However, while the results with the ΔIA double mutant were similar to those obtained with the ΔA single mutant, a cumulative effect could be observed in the ΔEA strain, which showed a significant decrease in colonization of both the whole root and the root tip. This phenotype was very similar to that observed in the triple mutant. In this set of experiments the Sm resistance marker seemed to confer a slight advantage over the Km resistance marker, according to the results obtained with the wild type derivatives tagged with each mini-Tn7 (Figure 6). This could suggest that the actual influence of the *rsm* mutations might be larger than observed here.

The loss of fitness in the rhizosphere of the ΔEA and ΔIEA mutants may result from the overall influence of Rsm proteins on different metabolic processes, including amino acid transport, while the reduction in root tip colonzation could correlate with the previously observed decrease in swimming motility in the ΔEA and ΔIEA strains and the lack of swarming motility of all the rsm mutants, with the exception of ΔI, which still retained some surface motility (Huertas-Rosales et al., 2016). It will also be of interest to explore whether the type VI secretion system K1, some of whose genes were identified in our study (Table 3), may contribute to the fitness of KT2440 in the rhizosphere. It has been reported that this system can provide a competitive advantage to this strain in the presence of phytopathogenic bacteria (Bernal et al., 2017).

## Concluding Remarks

This work represents the first effort to define the global regulatory network commanded by Rsm proteins in *P. putida*. Besides exposing its complexity and the vast influence that post-transcriptional regulation is bound to have in this bacterium, ranging from amino acid metabolism to potential transposon-mediated DNA rearrangements, the information obtained leads to a relevant question to be analyzed in detail, i.e. the characteristics by which an RNA molecule constitutes a shared target for the three Rsm proteins or is selectively bound by only one or two of them.

## Data Availability

The datasets presented in this study can be found in online repositories. The names of the repository/repositories and accession number(s) can be found in the article/Supplementary Material.

## References

[B1] Ausubel F. M. Brent R. Kingston R. E. Moore D. D. Seidman J. G. Smith J. A. (1987). Current protocols in molecular biology. New York, NY: Wiley.

[B2] Baker C. S. Eöry L. A. Yakhnin H. Mercante J. Romeo T. Babitzke P. (2007). CsrA inhibits translation initiation of *Escherichia coli hfq* by binding to a single site overlapping the Shine-Dalgarno sequence. J. Bacteriol. 189, 5472–5481. 10.1128/JB.00529-07 17526692 PMC1951803

[B3] Barrientos-Moreno L. Molina-Henares M. A. Ramos-González M. I. Espinosa-Urgel M. (2020). Arginine as an environmental and metabolic cue for cyclic diguanylate signalling and biofilm formation in *Pseudomonas putida* . Sci. Rep. 10 (1), 13623. 10.1038/s41598-020-70675-x 32788689 PMC7423604

[B4] Bernal P. Allsopp L. P. Filloux A. Llamas M. A. (2017). The *Pseudomonas putida* T6SS is a plant warden against phytopathogens. ISME J. 11, 972–987. 10.1038/ismej.2016.169 28045455 PMC5363822

[B5] Brencic A. Lory S. (2009). Determination of the regulon and identification of novel mRNA targets of *Pseudomonas aeruginosa* RsmA. Mol. Microbiol. 72, 612–632. 10.1111/j.1365-2958.2009.06670.x 19426209 PMC5567987

[B6] Brencic A. McFarland K. A. McManus H. R. Castang S. Mogno I. Dove S. L. (2009). The GacS/GacA signal transduction system of *Pseudomonas aeruginosa* acts exclusively through its control over the transcription of the RsmY and RsmZ regulatory small RNAs. Mol. Microbiol. 73, 434–445. 10.1111/j.1365-2958.2009.06782.x 19602144 PMC2761719

[B7] Choi K. H. Kumar A. Schweizer H. P. (2006). A 10-min method for preparation of highly electrocompetent *Pseudomonas aeruginosa* cells: application for DNA fragment transfer between chromosomes and plasmid transformation. J. Microbiol. Methods 64, 391–397. 10.1016/j.mimet.2005.06.001 15987659

[B8] Coggan K. A. Wolfgang M. C. (2012). Global regulatory pathways and cross-talk control *Pseudomonas aeruginosa* environmental lifestyle and virulence phenotype. Curr. Issues Mol. Biol. 14, 47–70. 22354680 PMC12747716

[B9] Dubey A. K. Baker C. S. Romeo T. Babitzke P. (2005). RNA sequence and secondary structure participate in high-affinity CsrA-RNA interaction. RNA 11, 1579–1587. 10.1261/rna.2990205 16131593 PMC1370842

[B10] Duss O. Michel E. Diarra dit Konté N. Schubert M. Allain F. H. (2014). Molecular basis for the wide range of affinity found in Csr/Rsm protein-RNA recognition. Nucleic Acids Res. 42, 5332–5346. 10.1093/nar/gku141 24561806 PMC4005645

[B11] Edwards A. N. Patterson-Fortin L. M. Vakulskas C. A. Mercante J. W. Potrykus K. Vinella D. (2011). Circuitry linking the Csr and stringent response global regulatory systems. Mol. Microbiol. 80, 1561–1580. 10.1111/j.1365-2958.2011.07663.x 21488981 PMC3115499

[B12] Espinosa-Urgel M. Ramos J. L. (2001). Expression of a *Pseudomonas putida* aminotransferase involved in lysine catabolism is induced in the rhizosphere. Appl. Environ. Microbiol. 67, 5219–5224. 10.1128/AEM.67.11.5219-5224.2001 11679348 PMC93293

[B13] Ferreiro M. D. Nogales J. Farias G. A. Olmedilla A. Sanjuán J. Gallegos M. T. (2018). Multiple CsrA proteins control key virulence traits in *Pseudomonas syringae* pv. tomato DC3000. Mol. Plant Microbe Interact. 31, 525–536. 10.1094/MPMI-09-17-0232-R 29261011

[B14] Figueroa-Bossi N. Schwartz A. Guillemardet B. D’Heygère F. Bossi L. Boudvillain M. (2014). RNA remodeling by bacterial global regulator CsrA promotes Rho-dependent transcription termination. Genes Dev. 28, 1239–1251. 10.1101/gad.240192.114 24888591 PMC4052769

[B15] Heeb S. Valverde C. Gigot-Bonnefoy C. Haas D. (2005). Role of the stress sigma factor RpoS in GacA/RsmA-controlled secondary metabolism and resistance to oxidative stress in *Pseudomonas fluorescens* CHA0. FEMS Microbiol. Lett. 243, 251–258. 10.1016/j.femsle.2004.12.008 15668026

[B16] Heroven A. K. Böhme K. Dersch P. (2012). The Csr/Rsm system of *Yersinia* and related pathogens: a post-transcriptional strategy for managing virulence. RNA Biol. 9, 379–391. 10.4161/rna.19333 22336760

[B17] Hinsa S. M. Espinosa-Urgel M. Ramos J. L. O’Toole G. A. (2003). Transition from reversible to irreversible attachment during biofilm formation by *Pseudomonas fluorescens* WCS365 requires an ABC transporter and a large secreted protein. Mol. Microbiol. 49, 905–918. 10.1046/j.1365-2958.2003.03615.x 12890017

[B18] Hör J. Gorski S. A. Vogel J. (2018). Bacterial RNA biology on a genome scale. Mol. Cell 70, 785–799. 10.1016/j.molcel.2017.12.023 29358079

[B19] Huertas-Rosales Ó. Ramos-González M. I. Espinosa-Urgel M. (2016). Self-regulation and interplay of Rsm family proteins modulate the lifestyle of *Pseudomonas putida* . Appl. Environ. Microbiol. 82, 5673–5686. 10.1128/AEM.01724-16 27422830 PMC5007770

[B20] Huertas-Rosales Ó. Romero M. Heeb S. Espinosa-Urgel M. Cámara M. Ramos-González M. I. (2017). The *Pseudomonas putida* CsrA/RsmA homologues negatively affect c-di-GMP pools and biofilm formation through the GGDEF/EAL response regulator CfcR. Environ. Microbiol. 19, 3551–3566. 10.1111/1462-2920.13848 28677348 PMC6849547

[B21] Ishihama A. (2010). Prokaryotic genome regulation: multifactor promoters, multitarget regulators and hierarchic networks. FEMS Microbiol. Rev. 34, 628–645. 10.1111/j.1574-6976.2010.00227.x 20491932

[B22] Jacob F. Monod J. (1961). Genetic regulatory mechanisms in the synthesis of proteins. J. Mol. Biol. 3, 318–356. 10.1016/s0022-2836(61)80072-7 13718526

[B23] Janssen K. H. Diaz M. R. Golden M. Graham J. W. Sanders W. Wolfgang M. C. (2018). Functional analyses of the RsmY and RsmZ small noncoding regulatory RNAs in *Pseudomonas aeruginosa* . J. Bacteriol. 200, e00736–17. 10.1128/JB.00736-17 29463606 PMC5952390

[B24] Jiménez-Fernández A. López-Sánchez A. Calero P. Govantes F. (2015). The c-di-GMP phosphodiesterase BifA regulates biofilm development in *Pseudomonas putida* . Environ Microbiol Rep 7, 78–84. 10.1111/1758-2229.12153 25870874

[B25] Kay E. Dubuis C. Haas D. (2005). Three small RNAs jointly ensure secondary metabolism and biocontrol in *Pseudomonas fluorescens* CHA0. Proc. Natl. Acad. Sci. U.S.A. 102, 17136–17141. 10.1073/pnas.0505673102 16286659 PMC1287983

[B26] Koch B. Jensen L. E. Nybroe O. (2001). A panel of Tn7-based vectors for insertion of the *gfp* marker gene or for delivery of cloned DNA into Gram-negative bacteria at a neutral chromosomal site. J. Microbiol. Methods 45, 187–195. 10.1016/s0167-7012(01)00246-9 11348676

[B27] Langmead B. Trapnell C. Pop M. Salzberg S. L. (2009). Ultrafast and memory-efficient alignment of short DNA sequences to the human genome. Genome Biol. 10, R25. 10.1186/gb-2009-10-3-r25 19261174 PMC2690996

[B28] Lawhon S. D. Frye J. G. Suyemoto M. Porwollik S. McClelland M. Altier C. (2003). Global regulation by CsrA in *Salmonella typhimurium* . Mol. Microbiol. 48, 1633–1645. 10.1046/j.1365-2958.2003.03535.x 12791144

[B29] Lennox E. S. (1955). Transduction of linked genetic characters of the host by bacteriophage P1. Virology 1, 190–206. 10.1016/0042-6822(55)90016-7 13267987

[B30] Lenz D. H. Miller M. B. Zhu J. Kulkarni R. V. Bassler B. L. (2005). CsrA and three redundant small RNAs regulate quorum sensing in *Vibrio cholerae* . Mol. Microbiol. 58, 1186–1202. 10.1111/j.1365-2958.2005.04902.x 16262799

[B31] Martínez-Gil M. Yousef-Coronado F. Espinosa-Urgel M. (2010). LapF, the second largest *Pseudomonas putida* protein, contributes to plant root colonization and determines biofilm architecture. Mol. Microbiol. 77, 549–561. 10.1111/j.1365-2958.2010.07249.x 20545856

[B32] Matilla M. A. Espinosa-Urgel M. Rodríguez-Herva J. J. Ramos J. L. Ramos-González M. I. (2007). Genomic analysis reveals the major driving forces of bacterial life in the rhizosphere. Genome Biol. 8, R179. 10.1186/gb-2007-8-9-r179 17784941 PMC2375017

[B33] Matilla M. A. Travieso M. L. Ramos J. L. Ramos-González M. I. (2011). Cyclic diguanylate turnover mediated by the sole GGDEF/EAL response regulator in *Pseudomonas putida*: its role in the rhizosphere and an analysis of its target processes. Environ. Microbiol. 13, 1745. 10.1111/j.1462-2920.2011.02499.x 21554519

[B34] Morris E. R. Hall G. Li C. Heeb S. Kulkarni R. V. Lovelock L. (2013). Structural rearrangement in an RsmA/CsrA ortholog of *Pseudomonas aeruginosa* creates a dimeric RNA-binding protein, RsmN. Structure 21, 1659–1671. 10.1016/j.str.2013.07.007 23954502 PMC3791407

[B35] Nilsson M. Chiang W. C. Fazli M. Gjermansen M. Givskov M. Tolker-Nielsen T. (2011). Influence of putative exopolysaccharide genes on *Pseudomonas putida* KT2440 biofilm stability. Environ. Microbiol. 13, 1357–1369. 10.1111/j.1462-2920.2011.02447.x 21507178

[B36] Pannuri A. Vakulskas C. A. Zere T. McGibbon L. C. Edwards A. N. Georgellis D. (2016). Circuitry linking the catabolite repression and Csr global regulatory systems of *Escherichia coli* . J. Bacteriol. 198, 3000–3015. 10.1128/JB.00454-16 27551019 PMC5055604

[B37] Petrova O. E. Cherny K. E. Sauer K. (2014). The *Pseudomonas aeruginosa* diguanylate cyclase GcbA, a homolog of *P. fluorescens* GcbA, promotes initial attachment to surfaces, but not biofilm formation, via regulation of motility. J. Bacteriol. 196, 2827–2841. 10.1128/JB.01628-14 24891445 PMC4135668

[B38] Ramos-González M. I. MA Matilla. Quesada J. M. Ramos J. L. Espinosa-Urgel M. (2013). “Using genomics to unveil bacterial determinants of rhizosphere life style,” in Molecular microbial ecology of the rhizosphere. Editor de Bruijn F. J. (Hoboken, NJ: John Wiley & Sons), Vol. 1, 7–16.

[B39] Ramos-González M. I. Travieso M. L. Soriano M. I. Matilla M. A. Huertas-Rosales Ó. Barrientos-Moreno L. (2016). Genetic dissection of the regulatory network associated with high c-di-GMP levels in *Pseudomonas putida* KT2440. Front. Microbiol. 7, 1093. 10.3389/fmicb.2016.01093 27489550 PMC4951495

[B40] Regenhardt D. Heuer H. Heim S. Fernández D. U. Strömpl C. Moore E. R. (2002). Pedigree and taxonomic credentials of *Pseudomonas putida* strain KT2440. Environ. Microbiol. 4, 912–915. 10.1046/j.1462-2920.2002.00368.x 12534472

[B41] Reimmann C. Valverde C. Kay E. Haas D. (2005). Posttranscriptional repression of GacS/GacA-controlled genes by the RNA-binding protein RsmE acting together with RsmA in the biocontrol strain *Pseudomonas fluorescens* CHA0. J. Bacteriol. 187, 276–285. 10.1128/JB.187.1.276-285.2005 15601712 PMC538806

[B42] Romeo T. (1998). Global regulation by the small RNA-binding protein CsrA and the non-coding RNA molecule CsrB. Mol. Microbiol. 29, 1321–1330. 10.1046/j.1365-2958.1998.01021.x 9781871

[B43] Romeo T. Vakulskas C. A. Babitzke P. (2013). Post-transcriptional regulation on a global scale: form and function of Csr/Rsm systems. Environ. Microbiol. 15, 313–324. 10.1111/j.1462-2920.2012.02794.x 22672726 PMC3443267

[B44] Romero M. Silistre H. Lovelock L. Wright V. J. Chan K. G. Hong K. W. (2018). Genome-wide mapping of the RNA targets of the *Pseudomonas aeruginosa* riboregulatory protein RsmN. Nucleic Acids Res. 46, 6823–6840. 10.1093/nar/gky324 29718466 PMC6061880

[B45] Sabnis N. A. Yang H. Romeo T. (1995). Pleiotropic regulation of central carbohydrate metabolism in *Escherichia coli* via the gene *csrA* . J. Biol. Chem. 270, 29096–29104. 10.1074/jbc.270.49.29096 7493933

[B46] Sambrook J. Russell D. W. (2001). Molecular cloning: a laboratory manual. New York, NY: Cold Spring Harbor Laboratory Press, Cold Spring Harbor.

[B47] Shis D. L. Bennett M. R. Igoshin O. A. (2018). Dynamics of bacterial gene regulatory networks. Annu. Rev. Biophys. 47, 447–467. 10.1146/annurev-biophys-070317-032947 29570353

[B48] Sonnleitner E. Haas D. (2011). Small RNAs as regulators of primary and secondary metabolism in *Pseudomonas* species. Appl. Microbiol. Biotechnol. 91, 63–79. 10.1007/s00253-011-3332-1 21607656

[B49] Sowa S. W. Gelderman G. Leistra A. N. Buvanendiran A. Lipp S. Pitaktong A. (2017). Integrative FourD omics approach profiles the target network of the carbon storage regulatory system. Nucleic Acids Res. 45, 1673–1686. 10.1093/nar/gkx048 28126921 PMC5389547

[B50] Sterzenbach T. Nguyen K. T. Nuccio S. P. Winter M. G. Vakulskas C. A. Clegg S. (2013). A novel CsrA titration mechanism regulates fimbrial gene expression in *Salmonella typhimurium* . EMBO J. 32, 2872–2883. 10.1038/emboj.2013.206 24056837 PMC3817462

[B51] Vakulskas C. A. Potts A. H. Babitzke P. Ahmer B. M. Romeo T. (2015). Regulation of bacterial virulence by Csr (Rsm) systems. Microbiol. Mol. Biol. Rev. 79, 193–224. 10.1128/MMBR.00052-14 25833324 PMC4394879

[B52] Van Assche E. Van Puyvelde S. Vanderleyden J. Steenackers H. P. (2015). RNA-binding proteins involved in post-transcriptional regulation in bacteria. Front. Microbiol. 6, 141. 10.3389/fmicb.2015.00141 25784899 PMC4347634

[B53] Winsor G. L. Griffiths E. J. Lo R. Dhillon B. K. Shay J. A. Brinkman F. S. (2016). Enhanced annotations and features for comparing thousands of *Pseudomonas* genomes in the *Pseudomonas* genome database. Nucleic Acids Res. 44 (D1), D646–D653. 10.1093/nar/gkv1227 26578582 PMC4702867

[B54] Xiao Y. Nie H. Liu H. Chen W. Huang Q. (2016). Expression of the diguanylate cyclase GcbA is regulated by FleQ in response to cyclic di-GMP in *Pseudomonas putida* KT2440. Environ Microbiol Rep 8, 993–1002. 10.1111/1758-2229.12478 27701843

[B55] Yakhnin A. V. Baker C. S. Vakulskas C. A. Yakhnin H. Berezin I. Romeo T. (2013). CsrA activates flhDC expression by protecting flhDC mRNA from RNase E-mediated cleavage. Mol. Microbiol. 87, 851–866. 10.1111/mmi.12136 23305111 PMC3567230

[B56] Yakhnin H. Pandit P. Petty T. J. Baker C. S. Romeo T. Babitzke P. (2007). CsrA of *Bacillus subtilis* regulates translation initiation of the gene encoding the flagellin protein (*hag*) by blocking ribosome binding. Mol. Microbiol. 64, 1605–1620. 10.1111/j.1365-2958.2007.05765.x 17555441

[B57] Yang H. Liu M. Y. Romeo T. (1996). Coordinate genetic regulation of glycogen catabolism and biosynthesis in *Escherichia coli* via the *csrA* gene product. J. Bacteriol. 178, 1012–1017. 10.1128/jb.178.4.1012-1017.1996 8576033 PMC177760

[B58] Yousef-Coronado F. Travieso M. L. Espinosa-Urgel M. (2008). Different, overlapping mechanisms for colonization of abiotic and plant surfaces by *Pseudomonas putida* . FEMS Microbiol. Lett. 288, 118–124. 10.1111/j.1574-6968.2008.01339.x 18783437

[B59] Zhang Y. Liu T. Meyer C. A. Eeckhoute J. Johnson D. S. Bernstein B. E. (2008). Model-based analysis of ChIP-seq (MACS). Genome Biol. 9, R137. 10.1186/gb-2008-9-9-r137 18798982 PMC2592715

