# Supplementary material for: Genome-Wide Analysis of Targets for Post-Transcriptional Regulation by Rsm Proteins in Pseudomonas putida
Source: Front Mol Biosci. 2021 Feb 22;8:624061. doi: 10.3389/fmolb.2021.624061 (PMC7937890; doi:10.3389/fmolb.2021.624061)
Supplement: Supplementary file 1 [file datasheet1.pdf]

SUPPLEMENTARY MATERIAL

Genome-wide analysis of targets for post-transcriptional regulation by Rsm proteins in *Pseudomonas putida*

Óscar Huertas-Rosales, Manuel Romero, Kok-Gan Chan, Kar Wai Hong, Miguel Cámara, Stephan Heeb, Laura Barrientos-Moreno, María Antonia Molina-Henares, María L. Travieso, María Isabel Ramos-González, Manuel Espinosa-Urgel

Tables S1. Targets of Rsm proteins in *P. putida* KT2440 above the indicated cutoff parameters. Start and end genome coordinates for each RNA species in the different biological replicas, and the average fold enrichment values are shown, along with the loci covered in each case. The number of targets indicated in the Venn diagram in Figure 2 is the number shown in square brackets in boldface, after removing redundancies (see notes). Loci orientation is indicated by > (+strand) and < (-strand). When the target includes sequences corresponding to more than one locus, the ones with the longest overlap are shown in boldface. Targets specifically mentioned in the text are shown in red. When long intergenic sequences are included, these are indicated in parentheses.

Colors indicate common targets as follows:

A+E+I
A+E
A+I
E+I

Notes:
a: 1 target in RsmE and RsmI; taken as 1 target in the Venn diagram
b: *IS*Ppu8 and the adjacent intergenic region are considered as 1 target in all cases
c: when 2 targets correspond to the same gene, they are taken as 1

**RsmA; -10logPval > 130; fold enrichment ≥ 2.15**					
RsmA1		RsmA2		average Fold	245 [**241**] targets
start	end	start	end	enr.	locus PP_
14181	15830	14354	15830	2.77	0013>
61840	62547	61839	62515	2.74	0053>
73718	74458	73729	74900	3.65	**0063>**<0064
94454	94930	94462	94939	3.09	0084>**<0085**
95034	95923	95028	95913	2.65	<0085 **86>87>**<88
147520	148125	147475	148138	3.12	**<0139**<0140
168416	170838	168416	170834	2.32	0158>0159>
171386	172939	171359	172941	6.21	16SA (rRNA)>
173232	176106	173071	176108	4.64	23SA (rRNA)>
176821	178369	176800	178370	7.40	5SA (rRNA)>
178673	181522	178511	181537	4.70	23SB (rRNA)>
225090	225629	225117	225618	4.41	<0173
253817	254334	253874	254318	3.15	<0203
313343	313943	313343	313945	2.26	**0258>**<0259
314667	315358	314708	315367	3.03	**0260>**0261>
333211	333697	333212	333676	3.64	5435>**0274>**
339227	340248	339227	340259	2.20	<0281**<0282 (*artJ*)**<0283
351734	352280	351724	352265	3.75	**0292>**0293>
355137	356457	355067	356454	2.34	<0295**<0296**
358290	360006	358257	360006	2.34	5441> **0298>**<0299

	371831	372650	378067	378783	2.51	0310>
	390819	391513	390819	391644	2.45	0325>
	394576	395854	394588	395840	2.34	0328>
	406290	408007	406290	408005	2.18	<0338
	432981	435388	432962	435392	2.37	<0356
a	448788	450486	448793	450452	3.61	0370>
a	450517	451034	450513	451049	8.36	**mr05 (*rsmY*)>**<0371
	524940	526464	524928	526469	6.07	16SC (rRNA)>
	526802	529660	526632	529683	4.43	23SC (rRNA)>
	593360	594184	593358	594171	2.67	0503>**<0504**
	627312	629948	627322	630285	2.27	**<0541<0542**<0543
	634152	635642	634126	635497	2.53	0546>
	641025	642518	641053	642923	2.56	<0552<0553
	655922	656371	655893	656381	4.42	<0563 (*gcbA*)
	672573	673831	672652	673856	2.32	0573>**<0574** <0575
	697826	699335	697803	699337	5.40	16SD (rRNA)>
	699875	702746	699861	702788	4.36	23SD (rRNA)>
	731735	733043	731795	733080	2.25	0625>
	734169	735287	734162	735289	2.84	<0626
	751774	752773	751762	752797	2.48	5451><0641
	792929	793689	792919	793688	2.16	<0678**<0679**
	826796	827530	826783	827496	5.13	0711>
	958559	959213	958536	959222	2.67	**0820>** 0821>
	1026884	1027651	1026650	1027643	2.37	<0884<0885
	1027877	1028824	1027878	1028799	2.24	<0885
	1048261	1048900	1048287	1048910	4.04	<0906**<0907**
	1084145	1084974	1084147	1084952	2.53	0938>**0939>**
	1103391	1104847	1103389	1104959	2.37	0964> 0965>
	1118683	1120218	1118677	1120244	2.17	0979>**0980>**
	1149910	1151059	1149882	1151081	2.32	<1009
	1158027	1158461	1158051	1158625	3.00	1015>
	1177549	1178761	1177513	1178772	3.52	1032>
	1223172	1224770	1223163	1224127	2.61	<1067**<1068**<1069
	1231802	1232687	1231405	1232644	2.29	<1073
	1242584	1242946	1242607	1242936	3.18	<1083
	1246975	1248211	1246994	1248212	2.29	1088>
	1284031	1285573	1284037	1285630	2.15	1121>1122>
	1316197	1316515	1316188	1316503	3.83	>mr_15
	1325493	1327014	1325484	1327035	5.98	16SE (rRNA)
	1327335	1330214	1327345	1330221	4.60	23SE (rRNA)
	1365307	1365890	1365301	1365866	2.95	<1189**<1190**<1191
	1388744	1389437	1388757	1389459	2.25	1210> 1211>
	1556616	1557427	1556722	1557248	4.16	1367>**5468>**<1368
	1566286	1567285	1566295	1567284	3.74	1375>
	1606506	1607728	1606526	1607758	2.44	<t64**<1408**
	1635349	1635782	1635348	1635764	3.64	1434>
	1642738	1643190	1642744	1643207	3.00	<1443
	1695927	1696463	1695966	1696447	4.18	1491>**1492>**
	1704467	1706089	1704431	1706144	2.50	1498>**1499>**1500>
	1707234	1708652	1707235	1708677	2.62	<1502<1503
	1712253	1712984	1712278	1712974	2.52	1506>
	1818338	1819856	1818329	1819847	2.20	1622>**1623 (*rpoS*)>**
	1821916	1822568	1821915	1822572	4.58	**mr22 (*rsmZ*)><1625**
	1835561	1836387	1835569	1836388	2.56	<1640
	1850565	1851738	1850560	1853204	2.30	1655> **1656 (*relA*)>**
	1854898	1859288	1854881	1859321	2.31	1659>1660>1661>
	1929953	1930336	1929937	1930305	3.59	<1728**<1729**
	1932610	1933737	1932610	1933722	2.48	<1732<1733
	1941417	1942423	1941431	1942411	2.48	1741>
	1942490	1944699	1942485	1944588	2.16	1742>1743>
	1957538	1959041	1957518	1959064	2.35	1754><1755
	1966194	1968002	1966189	1968117	2.42	**1762>**<1763
	2039861	2040498	2039851	2040504	3.22	1813><1814
	2063621	2064532	2063624	2064379	2.26	1840>**<1841**
b	2085085	2086270	2085069	2086173	4.89	1865>
b	2087614	2088164	2087586	2088178	4.84	intergenic 1865/66
	2126927	2127555	2126932	2127745	3.70	1887>
	2239977	2241299	2239978	2240657	2.55	1977>
	2250636	2253157	2250633	2253175	2.27	**1985>1986>**1987>
	2253577	2256019	2253653	2256015	2.19	1987>**1988>1989**
	2370904	2372080	2370860	2372134	2.68	2080>

2388216	2388755	2388247	2388753	2.29	<5502<mr31
2412233	2413404	2412355	2413528	4.19	2113>(419bp)2114>
2439257	2440414	2439264	2440284	2.65	2137>
2492000	2494667	2493440	2494708	2.17	2185>**2186><2187**
2501704	2502448	2501869	2502460	3.94	2195>
2507033	2508097	2507624	2508096	3.52	2200>**<2201**
2526550	2529551	2526877	2529510	3.05	2215>**16>17>(405bp)**18>
2530422	2530940	2530425	2530952	4.88	2218>**2219>**
2548691	2550200	2548661	2550209	5.59	16SF (rRNA)
2550746	2553605	2550754	2553649	4.46	23SF (rRNA)
2624383	2624965	2624379	2624961	2.99	**2296>**2297>
2630537	2633506	2631293	2633498	2.27	2302> **2303>** 2304>
2640062	2640698	2639754	2640704	3.56	<2310
2668138	2672391	2668152	2672546	2.46	2338>**<2339**
2676298	2677399	2676326	2677333	2.46	**2345>2346>**2347>
2742167	2743107	2742155	2743094	3.64	**2396>**2397>
2783732	2784188	2783699	2784134	3.32	2437>
2786580	2786930	2786570	2786954	3.46	<2438
2787003	2787631	2787011	2787626	3.03	2439>
2799214	2800222	2799184	2800274	2.45	<2453
2816104	2817163	2816115	2817121	2.18	2470>**71>72 >t44**
2819979	2820327	2819970	2820324	3.70	2473>
2850955	2851307	2850943	2851315	4.63	**2504>**5530>
2935929	2937830	2935909	2937783	2.18	2569>
3024219	3024876	3024184	3025008	3.68	**<2640**<2641
3035273	3036416	3035278	3036411	3.47	<2648
3054043	3054519	3053259	3054527	3.87	<2666
3070099	3070487	3070086	3070431	4.18	2680>
3070606	3071644	3070593	3071642	3.36	**2681>**2682>
3114613	3115207	3114729	3115093	4.49	2730>2731>
3247256	3247711	3247262	3247692	3.42	2842>
3258671	3260415	3258656	3260425	2.35	<2856<2857
3273526	3273991	3273532	3274043	3.05	2871>
3301631	3302368	3301651	3302366	2.52	2904>
3311205	3312297	3310791	3312308	2.49	2911> 2912>
3325245	3327294	3325259	3327282	2.62	2925><2926
3349120	3350921	3349096	3350928	2.19	2944>**2945>**
3400458	3401230	3400542	3401258	5.02	<3006**<3007**
3402085	3402549	3402122	3402559	4.60	3010>
3464260	3465435	3464255	3465436	2.42	<3078**<3079**<3080
3477948	3478706	3477928	3478709	2.32	<3088**<3089**
3494411	3495339	3494402	3495329	4.34	<3098<3099
3513711	3514123	3513671	3514139	4.47	3108>
3540612	3542076	3540793	3542115	2.45	3127>**3128>**<3129 (*pea*)
3543579	3544552	3543671	3544604	2.78	<3130
3549398	3552017	3549253	3552040	2.17	3133>**34>35>**36> (*pea*)
3554125	3555107	3554036	3554899	2.40	3138>3139>
3575447	3576499	3575446	3576480	2.15	<3156
3671710	3672101	3671708	3672080	3.65	3234>**3235>**
3703482	3703885	3703460	3703885	4.14	<5597 (250 pb)
3748674	3750421	3748718	3750420	3.11	<3311**<3312<3313<3314**
3761211	3761602	3761203	3761598	3.71	3325>
3841818	3844425	3841847	3844429	2.25	<3391**<3392**<3393
3889343	3890909	3889347	3890885	2.39	3432>**<3433<3434**
3924796	3925304	3924810	3925292	3.37	<3461**<5602**
4013159	4013712	4013161	4013721	2.87	mr44>
4022139	4023114	4022125	4023093	6.04	<3547(666bp) <3548
4061881	4062838	4061882	4062802	2.41	**3580>**<3581
4086523	4087494	4086513	4087487	2.22	3596 (*amaD*)>**3597>**
4114287	4114797	4114285	4114747	4.09	<3620
4144163	4145054	4144145	4145096	2.72	3648>**3649>**
4160099	4160858	4160146	4160882	2.59	**3662>**<3663
4184187	4184875	4184152	4184801	3.80	3680>**3681>**
4197055	4197785	4197048	4197814	4.04	<5620
4209784	4210288	4209898	4210335	3.20	3692>
4244409	4245111	4244630	4245148	2.99	<3721<3722
4272704	4273098	4272712	4273117	3.85	**3743>**<3744
4289421	4290858	4289279	4290161	3.04	<3761 (*cfcA*)
4295633	4296371	4295634	4296298	2.56	3765>
4302943	4304486	4303221	4304468	2.97	**<3775**<3776
4306075	4306926	4306072	4306929	3.00	<3778

	4321162	4321821	4321170	4321826	3.09	5629>
	4325479	4326936	4325485	4326948	2.15	3796>
	4329856	4330807	4329980	4330799	2.27	3801>3802>
	4357839	4358524	4357838	4358524	2.53	**3832(*rsmE*)>**<3833
	4415756	4416152	4415762	4416153	4.06	3901>
	4418225	4419212	4418122	4419200	3.18	3908><3909
	4458536	4458988	4458505	4459004	3.32	3952>(266bp)3953>
a	4472735	4473269	4472724	4473293	4.08	3962>(665bp)
	4473355	4474160	4473367	4474239	4.77	**3963>**<3964
	4520030	4521771	4520018	4521771	2.21	4010><4011
	4533735	4534643	4533742	4534641	2.47	4021>
	4583557	4584376	4583534	4584141	2.34	<4060
	4634949	4636282	4635010	4636299	2.56	<4098**<99<100**
	4643129	4644681	4643116	4644693	2.47	4108><4109
	4645433	4647635	4645696	4647631	2.51	4111>
	4679897	4680813	4680062	4680799	2.71	<4143
	4698169	4698690	4698164	4698682	4.18	<4158
	4712301	4712839	4712285	4712848	2.91	4171>
	4739415	4740721	4739432	4740824	2.28	4194>
	4765303	4767845	4765312	4767833	2.69	4217>
	4839441	4842928	4839431	4842902	2.52	4254>55>56>57>58
	4850156	4851247	4850154	4851293	2.19	**4265>** 4266>
	4855177	4855801	4855185	4855801	2.56	4270>
	4858749	4859210	4858705	4859220	3.00	<mr49
	4866695	4867460	4866720	4867443	3.02	4278>
b	4907783	4908137	4908163	4908682	4.46	<4317**(277bp)4318>**
	4909436	4910660	4909457	4910667	4.21	**4318>(477bp)<19**<20
	5015913	5017379	5015913	5017387	2.71	<4421
	5097178	5099284	5097222	5099274	2.25	<4487
	5101878	5103150	5101881	5103118	2.35	4490>4491>
	5157544	5158127	5157551	5158127	4.27	**4541>**4542>
	5158518	5158900	5158534	5158931	3.91	4542>
	5180332	5181109	5180378	5181100	3.75	4558>**<4559**<4560
	5189154	5189742	5188989	5189780	2.54	<4569**<4570** <4571
	5190285	5191413	5190284	5191443	2.51	<4571
	5237101	5237601	5237094	5237604	2.57	5700>
	5264221	5267124	5264217	5267154	2.36	<4640<4641
	5291833	5294427	5291753	5294407	2.17	<4666<4667
	5307693	5310555	5307688	5310568	5.02	23SG (rRNA)
	5311157	5312725	5311125	5312725	6.36	16SG (rRNA)
	5316056	5317136	5316069	5317160	2.48	<4678
	5325028	5325565	5325055	5325537	2.36	4685>mr52>
	5338209	5338748	5338204	5338748	3.93	mr53>
	5344571	5346807	5344587	5346814	2.22	4702>
	5351298	5351797	5351288	5351823	3.20	<4707
	5351979	5353807	5351953	5353858	2.15	<4708
	5389637	5390452	5389635	5390495	2.49	4738> tm01>
	5425060	5425437	5425082	5425440	3.85	4764>(147pb)
	5437583	5437900	5437606	5437900	8.19	<mr57
	5455583	5455992	5455589	5455993	3.13	<4792<4793
	5458826	5459596	5458716	5459551	3.79	**4794>4795>**4796>
	5483693	5484213	5483695	5484190	3.29	4822>
	5533169	5534481	5533192	5534480	2.44	<4866<4867
	5542726	5545081	5542713	5545108	2.15	<4874<75<76<77
	5554163	5555573	5554156	5555561	3.06	<4885**<4886<4887**
	5557919	5559481	5557921	5559500	2.19	<4889<4890
	5592478	5592875	5592472	5592909	3.47	4920><4921
	5633408	5634449	5633348	5634471	2.67	4947>
	5655623	5657257	5655630	5657211	2.73	<4965
	5667891	5669578	5667863	5669590	2.42	**mr59>4976>**4977>
	5674697	5675503	5674697	5675814	2.17	4980>**<4981**<4982
	5695783	5696600	5695784	5696591	2.75	4999>
	5699356	5700384	5699356	5700535	2.44	5003>
	5704485	5705484	5704480	5705477	2.46	**<5007**<5008
	5752026	5753474	5752008	5753472	2.16	5046>
	5801087	5801596	5801067	5801584	2.77	**<5079**
	5804855	5805371	5804834	5805349	3.54	**<5083** 5084>
	5821902	5823133	5821909	5823129	2.91	5098>**5099>**5100>
	5852162	5852598	5852172	5852593	4.09	<5129<5720
	5892769	5893243	5892789	5893262	3.16	<5167
	5896282	5896649	5896275	5896571	3.90	<5171

5912714	5914201	5912653	5914182	2.55	5184>
5919005	5919586	5919017	5919555	2.37	**5189**>5190>
5930846	5931762	5930842	5931780	2.67	<5198<5199
5936428	5937988	5936420	5938023	3.39	**5206**>5207>
5941844	5942543	5941988	5942556	3.73	<5209
5965490	5966129	5965507	5966108	2.29	5231>**<5232**
6027907	6029544	6027908	6029541	2.39	**<5278**
6060952	6061450	6060951	6061470	2.45	<5313
6114030	6115621	6114043	6115616	2.20	5364>**<5365**
6130950	6131902	6131168	6131913	3.26	<5379**<5732**<5380
6145339	6147414	6145376	6147403	2.36	<5389**<90<91<92**
6150919	6153185	6150923	6153156	2.21	**5395>5737>**5738

**RsmE; -10logPval > 170; fold enrichment ≥ 4**							
RsmE1		RsmE2		RsmE3		average Fold	270 [**261**] targets
start	end	start	end	start	end	enr.	locus PP_
48149	48591	48149	48605	48207	48615	9.49	<0043
61346	62727	61356	62750	61338	62726	6.99	0053>
73573	74445	73544	74458	73676	74440	6.90	**0063**><64
74489	74890	74489	74900	74459	74888	7.18	**<0064**<0065
94555	94940	94507	94961	94511	94940	8.58	0084>**<0085**
105204	106008	105182	106008	105204	106002	4.32	0100>
107617	108469	107624	108479	107615	108453	4.75	0101>**<5426**
108701	110556	108701	110643	108731	111154	5.45	**0103**>0104>
155538	155919	155549	155974	155543	155780	8.65	<0146
161031	162057	161043	162029	161032	162066	4.72	<0152 <0153
169635	170001	169598	170110	169626	169947	7.23	0159>
219026	220648	219039	220615	219193	220663	4.59	0168 (*lapA*)> **5431>**
224752	225652	224764	225633	224752	225680	4.70	<0173
233559	234386	233595	234393	233547	234374	5.40	0180>**<0181**<0182
236999	238376	236996	238288	237057	238405	5.01	0185>
300468	300862	300498	300836	300497	300865	6.18	<0245
344515	344969	344455	344936	344643	344945	8.75	<0285**<0286**<0287
351586	352596	351614	352597	351599	352495	7.81	**0292**>0293>
439710	440037	439696	440037	439716	440026	7.99	<0361
448800	451299	448799	451290	448829	451258	16.46	**0370>mr05 (*rsmY*)>**<0371
456576	457049	456578	457039	456570	457007	7.71	<0375<0376
470467	470964	470465	470989	470465	470969	6.03	<0386
506318	507068	506317	507069	506392	507069	4.86	0417>
514196	516151	514267	516103	514265	516118	5.47	<0426**<0427<0428**
583890	584224	583916	584232	583924	584253	5.75	0493>**0494>PP_t75**
606171	606790	606129	606693	606220	606665	5.46	0521>
627337	628104	627338	628131	627339	628117	5.08	**<0541** <0542
634324	635488	634336	635499	634598	635475	5.79	0546>
639535	640533	639565	640532	639590	640477	7.69	<0551
655517	656394	655515	656397	655650	656375	5.58	<0563 (*gcbA*)
666110	666526	666112	666557	666198	666495	8.38	0570>
672699	673923	672730	673929	672720	673900	4.86	**<0574** <0575
713481	714728	713483	714751	713475	714693	5.69	0604>**0605>**0606>
733825	735319	733840	735348	733787	735340	6.10	<0626
762618	763265	762625	763234	762668	763277	7.64	0654> 0655>
826797	827801	826757	827762	826806	827750	5.33	**0711**>0712>
914446	914993	914565	914991	914564	914964	8.21	0795>
921446	921928	921455	921912	921471	921943	6.61	<0802
958562	959221	958568	959203	958561	959204	5.24	**0820>** 0821>
970998	971731	971013	971741	970995	971722	4.78	0833>**0834>**0835>
974623	975264	974648	975269	974629	975203	5.34	0837>
1044972	1045340	1044968	1045336	1044992	1045338	5.88	0904>**0905>**<0906
1048152	1048793	1048342	1048810	1048303	1048730	6.94	<0906**<0907**
1057527	1058106	1057537	1058137	1057519	1058105	5.07	<0914 (*bifA*)
1074736	1075187	1074779	1075224	1074736	1075216	5.61	<0930<931**<932**
1084055	1084992	1084040	1084995	1084066	1084966	6.93	0938>0939>
1094728	1095477	1094744	1095453	1094694	1095479	4.83	<0952
1114905	1115445	1114900	1115441	1114913	1115395	5.56	intergenic <977
1124594	1125125	1124608	1125128	1124665	1125077	4.88	<0986
1136929	1142624	1136939	1142604	1136929	1142628	4.24	<0997 to <1002 (*arcD*)

	1157829	1159238	1157858	1159222	1157860	1159110	5.14	1015>
	1177879	1178776	1177839	1178752	1177881	1178749	5.40	1032>
c	1206705	1207093	1206781	1207138	1206818	1207091	6.51	<1058
	1208466	1209019	1208402	1209026	1208475	1209045	6.69	
	1231773	1232720	1231732	1232714	1231665	1232714	7.87	<1073
	1242583	1242868	1242643	1242938	1242580	1242915	7.38	<1083
	1271714	1273841	1272242	1273205	1271703	1273614	4.77	1112>**1113>**<1114<1115
	1316253	1317046	1316225	1317038	1316256	1317047	5.37	mr15> 1150>
	1365326	1365958	1365354	1366063	1365379	1365866	4.95	**<1189<1190**<1191
	1375697	1376718	1375624	1376326	1375879	1376651	7.93	1200><1201
	1388757	1389802	1388737	1389781	1388757	1389771	4.46	1210> 1211>
	1431327	1431818	1431361	1431833	1431349	1431833	6.72	1252>**<1253**
	1556633	1557505	1556715	1557248	1556715	1557272	6.81	1367>**5468>**<1368
	1565412	1565992	1565389	1565984	1565379	1565967	6.68	intergenic <1374
	1635110	1635746	1635328	1635794	1635103	1635747	7.33	1434>
c	1642293	1643454	1642296	1643446	1642529	1643432	8.16	<1443
	1643524	1644222	1643591	1644158	1643562	1644127	7.66	
	1647384	1647864	1647396	1647864	1647386	1647805	7.96	<1445
	1689622	1690917	1689394	1690899	1689425	1690893	4.90	<1486
	1696064	1696451	1696112	1696463	1696101	1696439	7.04	1492>
	1697782	1698669	1697823	1698680	1697834	1698691	7.45	1492>1493>
	1704013	1706085	1704018	1706086	1704020	1705656	7.10	1497>**1498>1499>**1500>
	1708765	1709219	1708762	1709207	1708778	1709174	5.82	<1504
	1719241	1720003	1719246	1720011	1719244	1720002	7.56	<1515
	1802749	1803303	1802767	1803299	1802765	1803309	4.90	1606>
	1821872	1822671	1821901	1822713	1821906	1822661	11.58	**mr22 (*rsmZ*)><1625**
	1853775	1855283	1853781	1855289	1854082	1855283	4.83	1657>**1658>(309bp)** 1659>
	1879235	1880240	1879235	1880135	1879237	1880133	7.21	1686><1687
	1918256	1920379	1918258	1920379	1918258	1919398	4.49	<1719
	1925714	1926983	1925667	1926958	1925714	1926963	5.11	<1726
	1929947	1930338	1929939	1930340	1929944	1930345	7.12	<1728**<1729**
	1941388	1942473	1941398	1944606	1941381	1942473	4.59	1741>
	2002851	2003805	2002852	2003800	2002822	2003760	6.05	1786>1787>
	2039016	2039511	2039039	2039509	2039086	2039493	9.29	<PP1811
	2039853	2040903	2039822	2040752	2039842	2040780	7.75	**1813><1814** 1815>
	2064720	2065326	2064723	2065322	2064717	2065313	7.54	<1841
b	2085285	2085639	2085331	2086328	2085333	2086323	9.70	1865>
	2086914	2087443	2086904	2087515	2086903	2087531	8.64	1865> intergenic
	2087627	2088157	2087606	2088166	2087634	2088157	12.79	intergenic 1865/66
	2096575	2097187	2096616	2097283	2096615	2097145	5.31	**1873>**1874>
	2126867	2127593	2126869	2127731	2126864	2127746	7.00	1887>
	2139538	2140070	2139566	2140054	2139553	2140038	4.72	<5481
	2239829	2240569	2239819	2240564	2239932	2240547	4.37	1977>
	2256031	2257100	2256023	2257080	2256043	2257061	7.57	1990>
	2257648	2258277	2257663	2258214	2257655	2258214	6.66	1990> + intergenic
	2285735	2286337	2285720	2286487	2285710	2286342	6.19	2014>
	2294282	2295486	2294286	2295452	2294307	2295440	6.93	2020>**2021>**
	2370706	2372115	2370844	2372141	2370703	2372157	6.07	2080>
	2411915	2413595	2411898	2413603	2411946	2413594	8.30	2113>(419bp)2114>
	2459815	2461187	2459773	2461163	2459834	2461105	4.22	<2152**<2153** 2154>
	2476724	2477175	2476715	2477138	2476742	2477175	7.13	2170>
	2501699	2502463	2501944	2502453	2501911	2502445	6.36	2195>
	2525408	2529732	2525423	2529728	2525550	2529718	5.73	2214>**2215>2216>2217>**
	2530263	2531001	2530298	2530980	2530284	2530980	7.81	2218>**2219>**
	2541547	2542313	2541521	2542314	2541548	2542299	4.53	2234>
	2584616	2585481	2584616	2585463	2584617	2585485	5.37	<2265
	2607496	2608233	2607487	2608223	2607498	2608226	8.71	2284>5514>
	2622586	2623232	2622591	2623237	2622607	2623260	5.26	5516>**5517>**
	2676273	2677329	2676299	2677265	2676274	2677335	5.79	**2345>2346>**2347>
	2687646	2688278	2687623	2688122	2687646	2688110	7.08	<2354**<2355**<2356
	2728175	2728563	2728230	2728573	2728229	2728551	6.46	2390>
	2742155	2743204	2742175	2743177	2742154	2743135	9.02	**2396>**2397>
	2783511	2784358	2783504	2784360	2783544	2784229	7.92	2437>
	2850940	2851341	2850964	2851374	2850945	2851328	10.04	**2504>**5530>
	2851512	2851951	2851536	2851951	2851515	2851864	6.94	5531>
	2853095	2853409	2853032	2853437	2853059	2853447	6.35	<2505
	2869316	2869827	2869322	2869828	2869321	2869801	7.92	2522>
	2925578	2926162	2925597	2926162	2925582	2926168	5.84	<2564
	2948632	2949185	2948671	2949182	2948664	2949159	6.79	<2582
	3009217	3009656	3009217	3009666	3009223	3009659	4.66	2629 (*bcs*)>
	3024254	3024875	3024228	3024913	3024233	3024862	6.66	**<2640**<2641

	3070666	3071140	3070652	3071096	3070661	3071097	8.29	**2681**>2682>
	3248449	3248981	3248421	3248985	3248458	3248983	6.30	2845>
c	3253499	3253863	3253484	3253857	3253493	3253868	6.19	<2851 **<2852**
	3254136	3254775	3254267	3254777	3254285	3254716	7.77	<2852
	3255790	3256476	3255532	3256673	3255792	3256446	5.74	<2853
	3297128	3297629	3297140	3297629	3297140	3297608	5.87	2899>**<2900**
c	3301626	3302421	3301626	3302395	3301626	3302389	6.21	2904>
	3302641	3303245	3302623	3303242	3302652	3303222	4.77	
	3326375	3327133	3326608	3327123	3326577	3327061	6.26	2925><2926
	3346002	3346526	3346013	3346523	3345956	3346510	4.36	<2942
	3346658	3347330	3346658	3347133	3346660	3347082	9.39	2943>
	3400535	3401224	3400557	3401198	3400556	3401226	8.63	<3006**<3007**
	3401353	3402584	3401336	3402590	3401358	3402591	9.27	3010>
	3411956	3412659	3411866	3412655	3411928	3412694	6.18	5551><3025
	3478007	3478712	3477951	3478708	3477951	3478702	6.15	<3088**<3089**
	3494414	3495016	3494424	3495020	3494484	3494987	10.72	<3098<3099
	3513733	3514231	3513731	3514166	3513747	3514158	8.68	3108>
	3515449	3516527	3515450	3516520	3515450	3516405	6.93	5576>
	3543601	3544512	3543770	3544517	3543645	3544456	8.86	<3130
	3546568	3547036	3546559	3547033	3546531	3547033	8.02	<3131
	3550018	3550745	3549962	3550656	3549996	3550707	5.41	3133>3134> (*pea*)
	3553965	3555299	3554043	3555273	3554010	3555280	8.15	3139>
	3575617	3576427	3575691	3576425	3575617	3576418	5.16	<3156
	3671289	3674517	3671271	3674528	3671288	3673992	5.08	3234>**3235>3236>3237>**
	3703490	3703887	3703464	3703886	3703484	3703882	8.55	<5597 (250 pb)
	3721040	3722556	3721053	3722553	3721053	3722554	4.79	**<3287<5598**
	3748687	3750377	3748705	3750362	3748641	3750346	7.51	<3311**<3312<3313<3314**
	3772373	3773266	3772383	3773293	3772380	3773303	5.13	3332> 3333>
	3825784	3826450	3825782	3826460	3825784	3826450	5.04	<3380 (601pb)
	3860372	3861240	3860358	3861109	3860383	3861077	7.00	**3412**>3413>
	3867122	3867690	3867075	3867711	3867117	3867642	4.68	3416>
	3933808	3934172	3933817	3934153	3933806	3934142	7.94	<3467
	3976895	3977308	3976934	3977335	3976904	3977335	6.58	<3507
	3996007	3996340	3996013	3996339	3996011	3996341	6.58	3523>
	3998679	3999161	3998747	3999123	3998795	3999138	7.27	<3527
	4002154	4002752	4002182	4002708	4002217	4002708	6.50	3530>
	4012728	4013666	4012717	4013670	4012868	4013687	6.49	mr44>
	4021583	4023285	4021597	4023282	4021671	4023109	13.33	<3547(66bp)<3548
	4061873	4062693	4061911	4062672	4061914	4062645	5.70	**3580**><3581
	4114292	4114846	4114328	4114835	4114277	4114778	10.16	<3620
	4159962	4160928	4160012	4160914	4160114	4160939	5.93	3662><3663
	4174621	4174987	4174638	4174990	4174621	4174962	7.96	<3674
	4184157	4184899	4184190	4184882	4184188	4184881	8.08	3680>**3681>**
	4197080	4197810	4197075	4197816	4197062	4197807	7.08	<5620
	4244221	4245458	4244400	4245484	4244362	4245372	6.40	<3721<3722
	4272711	4273115	4272688	4273114	4272717	4273112	9.41	**3743>**<3744
	4289631	4290121	4289610	4290122	4289587	4290106	6.27	<3761 (*cfcA*)
	4295582	4296289	4295590	4296273	4295585	4296294	5.95	3765>
	4303196	4304433	4303196	4304452	4303225	4304469	8.05	**<3775**<3776
	4306151	4306878	4306073	4306879	4306191	4306921	5.19	<3778
	4357790	4358788	4357840	4358790	4357840	4358789	5.25	**3832(*rsmE*)**><3833
	4361794	4364305	4361791	4364269	4361794	4364279	5.23	**3837><5634(434pb)**3839>
	4411884	4412596	4411865	4412571	4411865	4412590	4.99	**3986>**3987>
	4415769	4416120	4415782	4416125	4415779	4416125	9.15	3901>
	4416716	4417118	4416656	4417127	4416714	4417117	6.56	**<3904** 3905>
	4418296	4419165	4418411	4419154	4418286	4419151	8.09	3908><3909
	4448629	4448995	4448638	4448980	4448655	4448988	7.26	3945>
	4472726	4474262	4472726	4474267	4473311	4474227	9.42	3962>**(665bp)3963>**<3964
	4480623	4481209	4480649	4481242	4480637	4481209	10.77	3972>
	4514497	4515267	4514653	4515273	4514655	4515250	4.57	<4004
	4557609	4558023	4557660	4558012	4557669	4558024	7.70	**4044**><4045
	4580158	4580473	4580162	4580485	4579906	4580473	6.02	**<4058**<4059
	4595471	4596298	4595497	4596252	4595512	4596249	5.33	**4070**><4071
	4634770	4636323	4634839	4636268	4634881	4636304	5.92	<4098**<4099<4100**
	4636349	4637163	4636366	4637173	4636368	4637132	4.44	**<4101<4102**
	4643957	4644658	4643955	4644677	4643945	4644666	5.80	4108><4109
	4663886	4666034	4663812	4666049	4664944	4666028	5.57	4125>**4126>4127>**4128
	4685817	4686348	4685804	4686356	4685814	4686349	7.90	4147>
	4697323	4698086	4697592	4698091	4697612	4698071	6.90	**<4157**<4158
	4712193	4712973	4712313	4712853	4712303	4712829	7.15	4171>
	4721120	4722514	4720907	4723429	4721195	4722504	6.03	<4178<4179

	4752235	4752821	4752264	4752802	4752264	4752793	4.74	<4207
	4835427	4835893	4835441	4835884	4835455	4835906	4.76	<4249
	4855218	4855717	4855189	4855708	4855194	4855744	7.83	4270>
	4904820	4905208	4904820	4905345	4904823	4905425	8.91	<4312 **<4313**
b	4907692	4908138	4907714	4908748	4907733	4908141	9.87	<4317**(277bp)4318>**
	4909310	4910803	4909308	4910823	4909324	4910760	7.02	4318>**(477bp)<4319**<4320
	4951982	4953159	4951984	4953188	4951987	4953187	5.20	<4361**<4362**<4363
	4953857	4954762	4953898	4954798	4953879	4954753	6.04	<4363**<4364**
	4978832	4980180	4978875	4980172	4979097	4980169	5.74	<4384**<4385**<4386
	5042856	5043344	5042858	5043333	5042856	5043333	4.98	4447>4448>
	5075688	5076277	5075691	5076282	5075689	5076240	9.02	**<t57** <4469
	5079600	5080888	5079754	5080892	5079576	5080863	5.48	**<t53<t52<4472(*rsmA*)**<73
	5093608	5094427	5093623	5094433	5093643	5094435	6.14	<4482<4483 (*hisP*)
	5116912	5117573	5116912	5117612	5116930	5117591	4.79	4503>
	5157501	5158166	5157499	5158093	5157550	5158157	8.95	**4541>**4542>
c	5158516	5158898	5158518	5159043	5158554	5158816	6.92	4542>
	5159142	5159788	5159224	5159793	5159296	5159785	6.15	
	5180407	5181107	5180422	5181080	5180417	5181076	6.85	4558>**<4559**<4560
	5190296	5191437	5190301	5191415	5190281	5191429	5.31	<4571
	5204175	5204688	5204154	5204681	5204210	5204690	4.16	**4584>** 4585>
	5212154	5212846	5212175	5212766	5212125	5212850	7.71	**4593>**<4594
	5222314	5222770	5222300	5222769	5222314	5222788	4.93	<4602
	5249189	5249880	5249125	5249863	5249164	5249860	7.78	<4624
	5286903	5287334	5286909	5287302	5286923	5287306	5.61	<4659
	5316056	5317178	5316058	5317193	5316059	5317163	4.98	**<4678**<4679
	5325015	5325595	5325053	5325594	5325064	5325595	6.96	**4685>mr52>**<4686
	5328807	5329383	5329073	5329391	5328808	5329400	6.32	<4689 4690>
c	5347487	5348090	5347487	5348089	5347486	5348101	7.95	4704>
	5348439	5348904	5348461	5348849	5348459	5348843	5.26	
	5351310	5351809	5351346	5351807	5351345	5351773	5.55	<4707
	5372715	5373297	5372705	5373308	5372755	5373296	5.20	<4724 mr_55>
	5430426	5431255	5430428	5431256	5430427	5431256	6.79	<4770
	5435513	5436120	5435531	5436131	5435531	5436105	4.57	**4773>**4774>
	5455188	5456020	5455172	5456020	5455186	5456014	5.03	<4792 <4793
	5458625	5459635	5458703	5459627	5458708	5459623	8.46	**4794>4795>**4796>
	5483549	5484189	5483567	5484199	5483675	5484182	6.68	4822>
	5485839	5486710	5485928	5486701	5485957	5486701	6.20	**4823>** 4824>
	5488484	5489549	5488544	5489540	5488544	5489518	7.77	4824>
	5555087	5555544	5555121	5555548	5555071	5555540	9.03	<4887
	5592474	5592930	5592495	5592884	5592492	5592893	7.05	**4920>**<4921
	5613042	5613709	5612993	5613661	5613030	5613875	7.36	4933>
	5650689	5651253	5650700	5651278	5650704	5651301	7.02	<4959 (*cfcR*)
	5655640	5656486	5655635	5656478	5655640	5656449	4.87	<4965
	5664430	5664880	5664401	5664885	5664422	5664864	4.96	**<4972**<4973
	5681935	5682416	5681890	5682399	5681934	5682399	6.38	<4987**<4988**
	5695689	5696600	5695801	5696591	5695787	5696549	7.18	4999>
	5696759	5697883	5696742	5697527	5696781	5697512	7.14	**5000>**5001>
	5699355	5700267	5699365	5700267	5699373	5700269	6.67	5003>
	5759516	5760088	5759501	5760077	5759513	5760082	5.30	**<5053**<5054
	5781405	5782114	5781358	5782114	5781453	5782142	9.96	5067>
	5800674	5801620	5800939	5801617	5801002	5801593	7.13	<5078**<5079**<5080
	5804854	5805658	5804844	5805640	5804844	5805388	8.25	**<5083** 5084>
	5821870	5823163	5821870	5823130	5821882	5823130	6.23	5098>**5099>**5100>
	5845434	5846052	5845437	5846038	5845437	5846037	5.46	5123>**5124>**<5125
	5851948	5853716	5851948	5853746	5851956	5853647	8.12	<5129**<5720**
	5892753	5893273	5892758	5893281	5892799	5893297	8.00	<5167
	5896282	5897092	5896292	5896825	5896295	5896807	9.03	<5171
	5913275	5914200	5913521	5914199	5913544	5914201	5.49	5184>
	5923798	5924466	5923803	5924473	5923803	5924488	4.57	<5192
	5931034	5931696	5931018	5931717	5931092	5931685	4.97	<5199
	5941939	5942546	5941960	5942527	5941958	5942516	5.79	<5722 **<5209**
	5944819	5945783	5944889	5945774	5945016	5945773	5.81	<5212<5213
	5949196	5949739	5949231	5949706	5949253	5949781	4.87	5216>
	5954026	5954533	5954017	5954510	5954106	5954490	7.18	<5219
	5965491	5966145	5965501	5966159	5965491	5966113	5.81	5231>**<5232**
	5970884	5971338	5970847	5971314	5970847	5971319	7.13	<5236
	6002374	6003944	6002389	6003934	6002376	6003935	5.09	<5258 (*amaB*)
	6022664	6023510	6022674	6023471	6022757	6023471	5.58	5273><5274
	6060949	6061445	6060948	6061430	6060950	6061427	5.27	<5313
	6064271	6065040	6064272	6065067	6064272	6065052	4.91	5318>**<5319**
	6079485	6080187	6079475	6080203	6079486	6080176	4.81	**5331>** 5332>

6081316	6081857	6081320	6081842	6081446	6081842	5.45	<5334**<5333**
6089350	6089939	6089348	6089937	6089342	6089970	5.37	5341>
6113605	6114372	6113553	6114376	6113558	6114235	6.53	5364><5365
6128212	6128573	6128200	6128610	6128198	6128561	7.71	intergenic 920 bp
6130955	6131951	6130916	6131949	6130948	6131951	9.63	<5379**<5732**<5380
6134313	6134853	6134329	6134827	6134306	6134851	10.43	<5382
6150907	6152899	6150903	6152902	6150908	6152882	4.51	**5395>5737>**5738

RsmI; -10logPval > 145; fold enrichment ≥ 2.5					
RsmI1		RsmI2		average Fold enr.	209 [**206**] targets
start	end	start	end		locus PP_
23967	25403	24003	25339	2.71	0018>0019>0020>
60863	61258	60870	61704	3.33	0052>
61838	62630	61830	62711	3.68	0053>
73521	74926	73541	75427	7.62	**0063>**<64
94468	94936	94483	94954	4.70	0084>**<0085**
95600	96671	95639	96670	3.66	<0088
108692	111857	108713	111926	2.90	**0103>**0104>
112014	113302	111962	113288	2.73	106><5427
146916	148103	146951	147385	3.56	**0138>**<0139
155511	155919	155504	155921	5.16	<0146
168426	169396	169598	169940	3.56	0158>159>
171363	172942	171080	172941	7.16	16SA (rRNA)>
173180	176096	173072	176142	6.66	23SA (rRNA)>
176804	178370	176522	178371	7.28	5SA (rRNA)>
178610	181527	178488	181523	6.84	23SB (rRNA)>
224781	225650	225006	225629	3.78	<0173
233489	234200	233442	234211	3.20	0180>**<0181**<0182
253825	254214	253820	254221	4.05	<0203
351543	352649	351539	352654	5.73	**0292>**0293>
355003	356480	354985	356504	2.71	<0295**<0296**
358292	359323	358292	359266	2.99	0298>
369410	372740	368898	372740	3.11	0308>**0309>0310>**
389257	394363	389206	394394	3.33	0325>**0326>0327>**
432998	435305	432972	435361	2.84	<0356
448800	451021	448830	450490	6.88	**0370>mr05 (*rsmY*)>**
506298	507117	506267	507099	4.11	0417>
524920	526504	524635	526501	6.10	16SC (rRNA)>
526763	529660	526617	529695	5.91	23SC (rRNA)>
579158	580848	579152	580797	2.94	0491>**0492>**
606159	606942	606167	606944	3.64	0521>
634629	635239	634444	635110	3.40	0546>
637745	638820	637768	638826	2.89	0549><0550
639529	639963	639514	639989	3.56	<0551
655567	656395	655305	656398	3.28	<0563 (*gcbA*)
666033	666686	666067	666728	3.37	0570>
694148	695862	694146	695865	3.87	**0596>**0597>
695982	696580	695917	696503	2.99	0597>
697805	699339	697410	699378	6.49	16SD (rRNA)>
699863	702795	699706	702756	6.55	23SD (rRNA)>
733840	735320	733825	735320	3.82	<0626
751809	752789	751783	752786	2.83	5451><0641
761997	762541	762137	762601	3.61	0654>
826787	828406	826751	828493	4.08	**0711>0712>**0713>
974625	975229	974647	975226	3.22	0837>
1048107	1048912	1048107	1048961	5.65	<0906**<0907**
1158064	1158426	1158081	1158462	3.82	1015>
1177974	1178719	1177913	1178702	3.52	1032>
1231808	1232663	1231780	1232678	5.15	<1073
1319736	1320680	1319745	1320824	2.72	1153>**<1154**
1325380	1327058	1325218	1327069	6.04	16SE (rRNA)
1327188	1330211	1327172	1330224	6.29	23SE (rRNA)
1360097	1361988	1360083	1362061	2.86	**1185>** 1186>1187>
1375793	1376670	1376112	1376710	4.47	1200><1201
1388724	1389812	1388759	1389808	2.81	1210> 1211>
1425348	1425658	1425367	1425701	4.76	1249>
1495689	1496512	1495624	1496449	2.71	1308>
1537939	1538635	1537797	1538628	3.42	**<1349**<1350
1566326	1567319	1566340	1567447	5.01	1375>

	1573953	1575015	1573939	1575028	2.65	1380>1381><1382
	1635320	1635790	1635189	1635769	3.90	1434>
	1642282	1644701	1642277	1644696	3.96	<1443
	1644892	1647302	1644901	1646419	3.74	<1444
	1685779	1686695	1685439	1686778	2.73	<1481
	1695940	1696516	1695818	1696569	4.39	1491>**1492>**
	1698159	1698700	1697845	1698751	3.36	1493>
	1704616	1706263	1704618	1706330	3.30	**1499>**1500>
	1707256	1708661	1707253	1708667	3.24	<1502<1503
	1719203	1720054	1719203	1720034	5.83	<1515
	1766446	1766896	1766442	1766876	4.72	1577>
	1787451	1789007	1787625	1789006	2.76	1593>**1594>**1595>
	1835623	1836312	1835623	1836310	3.42	<1640
	1854933	1859168	1854945	1859160	3.63	1659>1660>1661>
	1879213	1880317	1879236	1880201	4.70	1686><1687
	1897608	1898106	1897477	1898124	4.32	<1702
	1925665	1926965	1925679	1926966	2.73	<1725**<1726**
	2026484	2026992	2026412	2026905	4.12	1803>
	2031362	2032293	2031433	2032300	3.76	<1807
	2039853	2040522	2039849	2040507	4.25	1813><1814
b	2085772	2086410	2085769	2086417	5.52	1865>
	2086888	2087544	2086864	2087554	6.01	1865>intergenic 65-66
	2087547	2088172	2087638	2088121	7.35	intergenic 1865/66
	2126850	2127813	2126859	2127817	5.30	1887>
	2139514	2140075	2139519	2140075	3.12	<5481
	2256526	2257175	2256525	2257211	4.39	1990>
	2285735	2286220	2285755	2286309	3.52	2014>
	2289149	2289956	2289149	2290392	3.00	2017>
	2294300	2295443	2294316	2295448	3.39	2020>**2021>**
	2369092	2372135	2369528	2372134	3.34	2080>
	2411880	2413717	2411899	2413692	4.46	2113>(419bp)2114>
	2501701	2502374	2501697	2502511	3.64	2195>
	2506944	2507385	2507552	2508061	3.86	<2201
	2524966	2529815	2523877	2529813	4.14	**2214>15>16>17>(405bp)**18>
	2530270	2530961	2530281	2530933	4.94	2218> 2219>
	2548659	2550241	2548412	2550242	6.28	16SF (rRNA)
	2550729	2553646	2550572	2553644	6.41	23SF (rRNA)
	2584610	2585448	2584616	2585468	3.21	<2265
	2657642	2658090	2657641	2658313	3.46	<2330
	2668194	2672333	2668184	2672345	2.89	2338>**<2339**
	2676326	2677230	2676334	2676971	2.77	**2345>2346>**2347>
	2742170	2743129	2742175	2743181	5.45	**2396>**2397>
	2755088	2755490	2755077	2755531	4.87	2410>
	2783482	2784338	2783504	2784709	5.75	2437>
	2788800	2789237	2788800	2789246	4.59	2440>
	2869286	2869897	2869311	2869921	5.40	2522>
	2939506	2940482	2939514	2940481	3.38	<2572<2573
	3024234	3024879	3024139	3024908	4.41	**<2640**<2641
	3070651	3071192	3070649	3071194	5.36	**2681>**2682>
	3114664	3115259	3114730	3115191	5.12	2730>**2731>**
	3248430	3248960	3248467	3248992	4.11	2845>
	3254217	3254804	3254070	3254900	5.65	<2852
	3326681	3327240	3326574	3327253	3.87	2925>**<2926**
	3344589	3346538	3344581	3346531	3.11	<2941**<2942**
	3349590	3350687	3349633	3350897	3.23	2945>
	3387198	3388080	3387196	3387881	5.47	2989>
	3400573	3401194	3400579	3401192	4.40	<3007
	3401366	3401832	3401371	3401838	3.53	**3008>** 3009>
	3402134	3402574	3402157	3402555	5.32	3010>
	3412013	3412429	3411976	3412429	3.68	<3025
	3450153	3450659	3450168	3450655	3.30	<5558
	3455188	3457239	3454768	3457257	2.71	<3071**<3072**<3073
	3477468	3478904	3477456	3478928	3.88	<3088**<3089**<3090
	3487605	3489385	3487392	3489413	3.50	<3093**<3094**<3095
	3494351	3496818	3494331	3497550	2.61	<3098**<99**<100<5562
	3513180	3514941	3513107	3514865	3.86	3108>
	3543556	3544548	3543506	3544548	4.67	<3130
	3553994	3555353	3553974	3555346	3.75	3139>
	3575570	3576428	3575494	3576454	2.96	<3156
	3623243	3623667	3623152	3623656	5.04	<3194
	3671296	3674497	3671302	3673871	3.42	3234>**35>36>37>**

	3721182	3722529	3721203	3722554	3.52	**<3287<5598**
	3724863	3726626	3724899	3726637	2.90	3290>3291>
	3748560	3750347	3748613	3750383	4.95	<3311 **<12** <13<14
	3761043	3761588	3761088	3761587	5.23	3325>
	3860392	3861274	3860375	3861295	3.88	**3412**>3413>
	3907573	3908322	3907578	3908323	4.85	3448>**3449**>3450>
	3924820	3925303	3924796	3925278	3.89	<5602
	3976822	3977329	3976637	3977333	5.12	<3506<3507
	3995989	3996368	3995974	3996362	5.47	3523>
	4013152	4013676	4013149	4013687	3.22	mr44>
	4021953	4023071	4022177	4023038	8.69	<3547(66bp)<3548
	4061890	4062740	4061883	4062751	3.02	3580><3581
	4114268	4114850	4114268	4114908	5.83	<3620
	4144211	4144801	4144218	4145057	3.06	3648>3649>
	4160223	4160901	4159968	4160900	2.89	3662><3663
	4197115	4197615	4197112	4197713	3.05	intergenic 3688/89(2491bp)
	4289586	4290154	4289565	4290203	4.41	<3761 (*cfcA*)
	4295638	4296256	4295610	4296253	2.84	3765>
	4321156	4321834	4321158	4321821	4.59	5629>
	4357787	4358768	4357778	4358768	3.31	**3832(*rsmE*)>**<3833
	4361866	4362796	4361872	4364332	3.65	**3837**><5634
	4415759	4416133	4415758	4416122	5.40	3901>
	4416654	4417141	4416654	4417141	4.43	**<3904** 3905>
	4418396	4419146	4418411	4419150	4.53	3908><3909
	4473398	4473876	4473369	4473873	4.64	3963>
	4480654	4481212	4480634	4481192	6.49	3972>
	4579730	4580515	4579265	4580527	3.33	<4058
	4595485	4596280	4595477	4596313	3.25	**4070**><4071
	4635023	4636284	4635003	4636281	3.42	<4098**<4099<4100**
	4858679	4859133	4858717	4859178	3.01	<4274
	4866696	4869935	4866743	4869961	3.01	4278>
b	4908155	4908873	4908165	4908877	5.20	4318>
	4909254	4910951	4909278	4910944	5.27	4318>**(477bp)<4319**<4320
	4925086	4925614	4925089	4925613	3.78	<4335
	4951699	4953172	4951700	4953611	2.79	<4361**<4362**<4363
	4967880	4970341	4967838	4970352	2.60	<4377<4378
	4980609	4981317	4980596	4981329	2.81	<4388
	5075733	5076242	5075728	5076218	3.35	**<t63** <4469
	5106961	5107380	5106564	5107716	3.50	<4493
	5157625	5158095	5157640	5158129	4.15	**4541**>4542>
	5158520	5159782	5158479	5159091	4.03	4542>
	5180389	5181067	5180388	5181120	4.20	4558>**<4559**<4560
	5190292	5191418	5190321	5191416	2.92	<4571
	5212176	5212809	5212169	5212821	4.26	**4593**><4594
	5291515	5294442	5291460	5294429	2.88	4665>**<4666<4667**
	5307697	5310592	5307694	5310725	6.63	23SG (rRNA)
	5311130	5312800	5311161	5312974	5.64	16SG (rRNA)
	5324996	5325589	5325054	5325584	4.72	**4685>mr52**><4686
	5338217	5338729	5338211	5338727	3.53	mr53>
	5344626	5345714	5344603	5345517	2.76	4702>
	5367495	5371301	5367483	5371290	2.67	4720>**<4721<22**<23
	5425083	5425447	5425094	5425466	4.82	4764>
	5430406	5431243	5430411	5431232	3.57	<4770
	5455467	5456065	5455484	5456045	3.43	<4792**<4793**
	5458640	5459614	5458459	5459608	5.19	4794>**4795**>4796>
	5488472	5489411	5488425	5489432	4.14	4824>
	5523930	5524879	5523934	5524861	5.18	<4857
	5531175	5531611	5531189	5531656	3.75	<4865
	5546093	5548317	5546083	5548332	2.66	<4879<4880
	5592473	5592891	5592516	5592913	3.85	4920>
	5650120	5651262	5650118	5651260	3.34	<4959 (*cfcR*)
	5681741	5682662	5681666	5682432	4.05	<4987<4988
	5696718	5698771	5696734	5698791	2.77	5000>5001>
	5699381	5700330	5699384	5700383	2.70	5003>
	5704537	5705800	5704537	5705777	2.73	<5007<5008
	5781524	5782099	5781285	5782100	5.22	5067>
	5800868	5801619	5800867	5801606	4.66	<5078**<5079**<5080
	5804820	5805476	5804839	5805381	5.11	**<5083** 5084>
	5821823	5823516	5821799	5823564	5.00	5098>**5099**>5100>
	5851906	5852628	5852185	5852620	5.36	<5129**<5720**
	5930842	5931805	5930818	5932048	3.10	<5199

5936420	5938634	5936428	5938646	2.81	**5206>**5207>
5941863	5942565	5941870	5942550	4.40	<5722 **<5209**
5944727	5945874	5944741	5945987	3.95	<5212<5213
5953853	5954775	5953560	5954890	4.53	5218><5219
5965223	5966113	5965282	5966146	3.70	5231>**<5232**
6002366	6003938	6002357	6003925	2.76	<5258 (*amaB*)
6047749	6048206	6047729	6048230	4.13	5300>
6113477	6114553	6113522	6114537	5.54	5364><5365
6150911	6152846	6150950	6152880	2.62	**5395>5396>**5397>

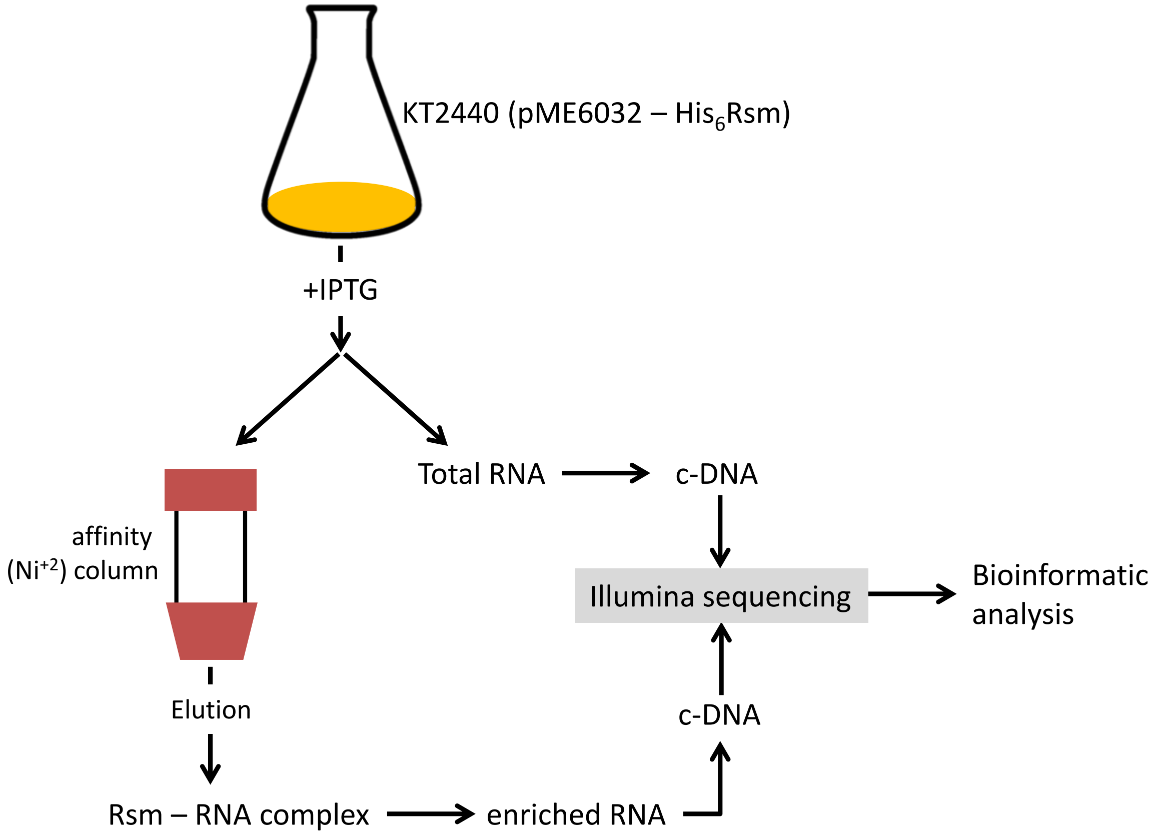

Figure S1. Schematic representation of the method used to identify Rsm-bound RNA sequences. See text for details.

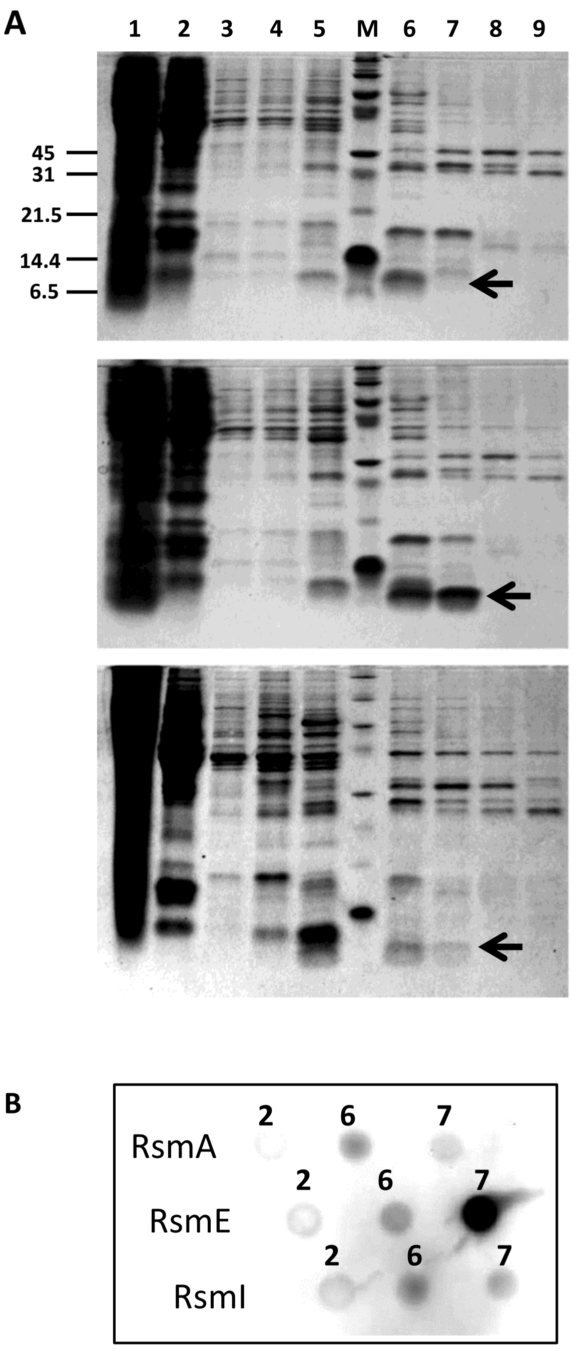

Figure S2. A. SDS-PAGE of fractions recovered by affinity purification of His_6-Rsm proteins from IPTG-induced KT2440 cultures harboring pME6032-*rsmA* (top) pME6032-*rsmE* (middle), and pME6032-*rsmI* (bottom). Lanes: 1, samples before injection in HisTrap columns (Amersham Bioscience); 2, elution with 7% imidazole; 3-9, fractions recovered in the 7-100% range of imidazole concentrations. Arrows indicate the bands possibly corresponding to Rsm proteins. **B.** Detection of His_6-Rsm proteins by dot blot. Twenty µL of the indicated samples (equivalent to the lanes in A) were spotted on an activated PVDF membrane and a monoclonal His-tag antibody (Invitrogen), diluted 1/5000 was used for chemiluminescence detection in a Bio-Rad ChemiDoc with ECL (Pierce) after incubation with a HRP-conjugated secondary antibody.

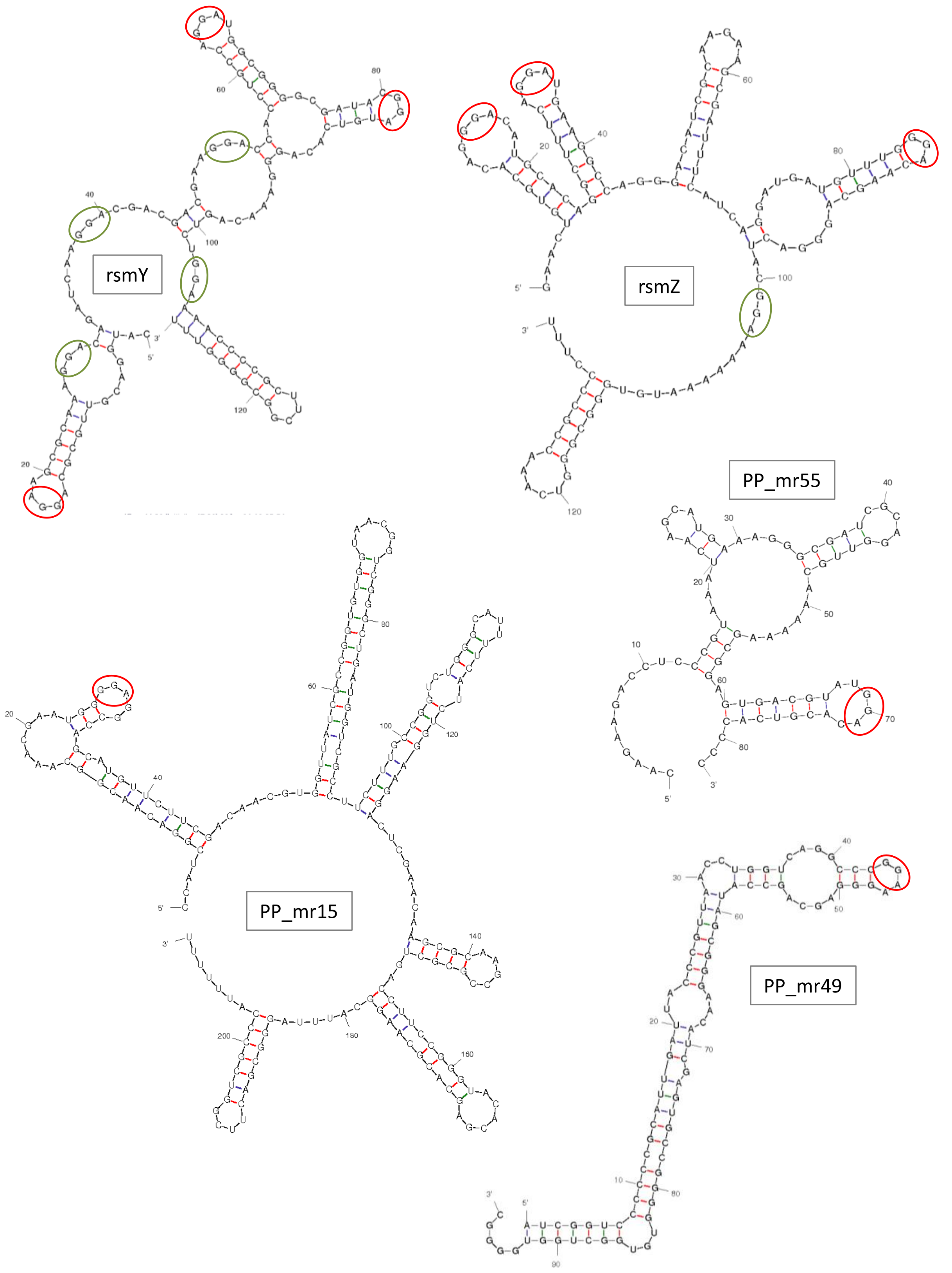

Figure S3. Secondary structure predictions for several ncRNAs of *P. putida*. Unpaired GGA motifs and those in stem-loop structures are indicated in green and red, respectively. Predictions were done using the Mfold web server (M. Zuker. Mfold web server for nucleic acid folding and hybridization prediction. *Nucleic Acids Res.* **31 (13)**, 3406-3415, 2003).

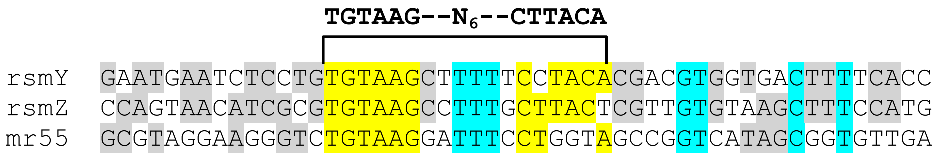

Figure S4. Putative GacA binding sites in the promoter regions of rsmY, rsmZ and PP_mr55. Bases identical to the consensus (indicated above the sequences) are highlighted in yellow; additional ones conserved in the three sequences are highlighted in blue and those conserved in two of the sequences in grey.

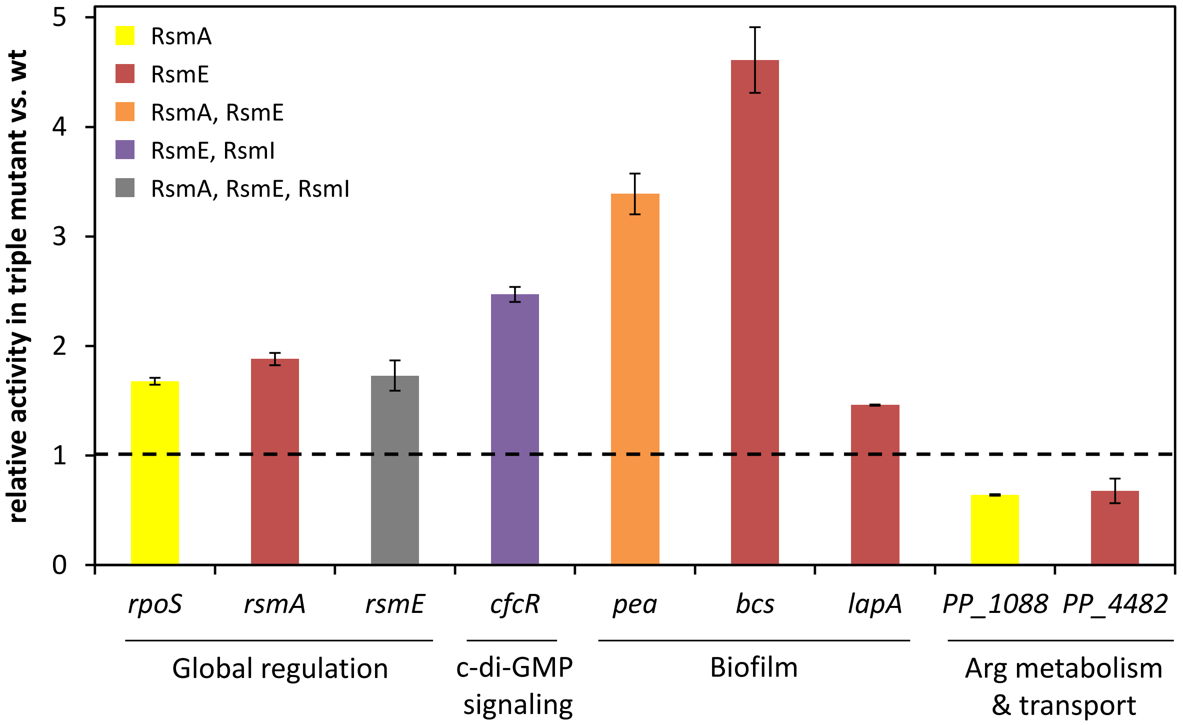

Figure S5. Influence of Rsm proteins on expression of some of the identified targets. Translation fusions to the reporter *'lacZ* were analyzed in KT2440 and the triple *rsm* mutant after 5h of growth. Data are presented as relative β-galactosidase activity in the mutant with respect to the wild type, and are averages and standard deviations of two independent experiments with three technical replicas each. A value of 1 (broken line) indicates that there is no difference between the two strains. Color codes indicate the Rsm protein(s) for which each RNA has been identifed as target. Constructs have been described elsewhere (Martínez-Gil et al., 2010; Huertas-Rosales et al., 2016, 2017; Barrientos-Moreno et al., manuscript in preparation).

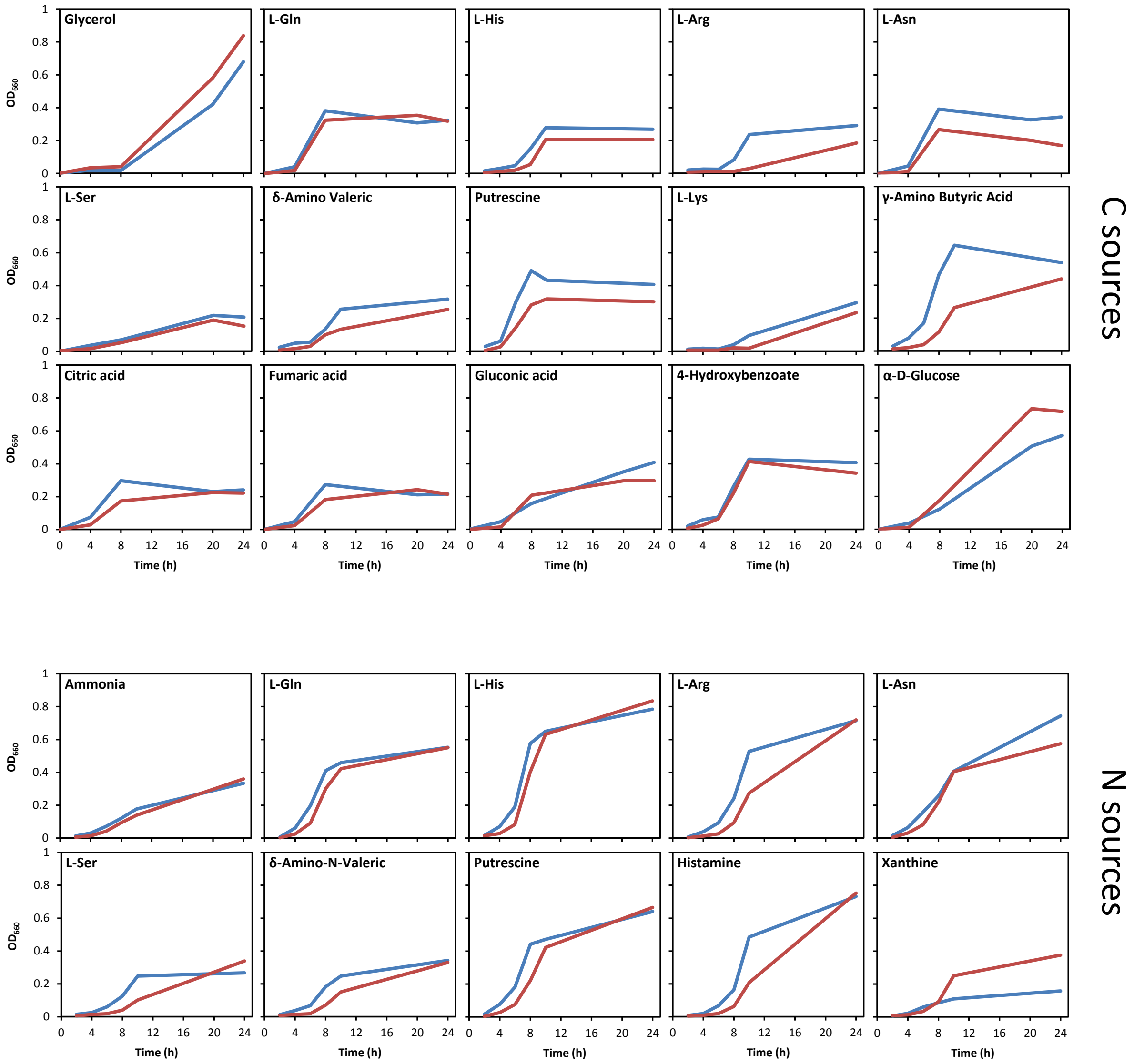

Figure S6. Growth of *P. putida* KT2440 (blue lines) and the triple *rsm* mutant (red lines) in BIOLOG plates during 24h of incubation at 30°C, with some of the compounds tested as sole source of carbon (top graphs) or nitrogen (bottom graphs).
